# IMM-BCP-01, a patient-derived anti-SARS-CoV-2 antibody cocktail, is active across variants of concern including Omicron BA.1 and BA.2

**DOI:** 10.1126/sciimmunol.abl9943

**Published:** 2022-06-30

**Authors:** Pavel A. Nikitin, Jillian M. DiMuzio, John P. Dowling, Nirja B. Patel, Jamie L. Bingaman-Steele, Baron C. Heimbach, Noeleya Henriquez, Chris Nicolescu, Antonio Polley, Eden L. Sikorski, Raymond J. Howanski, Mitchell Nath, Halley Shukla, Suzanne M. Scheaffer, James P. Finn, Li-Fang Liang, Todd Smith, Nadia Storm, Lindsay G. A. McKay, Rebecca I. Johnson, Lauren E. Malsick, Anna N. Honko, Anthony Griffiths, Michael S. Diamond, Purnanand Sarma, Dennis H. Geising, Michael J. Morin, Matthew K. Robinson

**Affiliations:** ^1^ Immunome, Inc., Exton, PA, U.S.A.; ^2^ Department of Microbiology, Boston University School of Medicine and National Emerging Infectious Diseases Laboratories, Boston, MA, USA; ^3^ Departments of Medicine, Molecular Microbiology, Pathology & Immunology, Washington University School of Medicine, St. Louis, MO 63110, USA

## Abstract

Monoclonal antibodies are an efficacious therapy against SARS-CoV-2. However, rapid viral mutagenesis, led to escape from most of these therapies, outlining the need for an antibody cocktail with a broad neutralizing potency. Using an unbiased interrogation of the memory B cell repertoire of convalescent COVID-19 patients, we identified human antibodies with broad antiviral activity in vitro and efficacy in vivo against all tested SARS-CoV-2 variants of concern, including Delta, Omicron BA.1 and BA.2. Here, we describe an antibody cocktail IMM-BCP-01, that consists of three patient-derived broadly neutralizing antibodies directed at non-overlapping surfaces on the SARS-CoV-2 spike protein. Two antibodies, IMM20184 and IMM20190, directly blocked Spike binding to the ACE2 receptor. Binding of the third antibody, IMM20253, to its cryptic epitope on the outer surface of RBD, altered the conformation of the Spike Trimer, promoting release of Spike monomers. These antibodies decreased Omicron SARS-CoV-2 infection in the lungs of Syrian golden hamsters in vivo, and potently induced antiviral effector response in vitro, including phagocytosis, ADCC, and complement pathway activation. Our pre-clinical data demonstrated that the three antibody cocktail IMM-BCP-01 could be a promising means for preventing or treating infection of SARS-CoV-2 variants of concern, including Omicron BA.1 and BA.2, in susceptible individuals.

## Teaser

IMM-BCP-01 is efficacious against Omicron, induces dissociation of Spike protein and triggers a potent effector response.

## INTRODUCTION

With over 527 million cases and more than 6.2 million deaths worldwide (Johns Hopkins University Coronavirus Resource Center), the SARS-CoV-2 pandemic continues to pose extraordinary health and economic challenges. The scientific community has mitigated this threat through the discovery and launch of a myriad of vaccines and therapeutics to prevent or treat infections. While the initial data for the original spike (S) protein-directed vaccines have been impressive, the current rate of protection against variants of concern is decreasing, which was predicted to occur due to viral escape and patient immunodeficiency or immunosuppression (*1*–*6*).

As such, the discovery and development of effective antibody therapies for passive immunization with broad range of reactivity is likely to be an important alternative approach to vaccination. The use of convalescent plasma against SARS-CoV-2 initially has yielded mixed results (*7*, *8*), however a recent retrospective cohort study showed reduced mortality in treated patients (*9*) outlining the need for a more robust and more standardized antiviral antibody cocktail. A phase 3 clinical trial with Lilly’s Bamlanivimab was halted on the basis of data showing no improvement in clinical outcomes. An early Regeneron trial with a 2-antibody mixture was also paused based on a potential safety signal and an unfavorable risk/benefit profile (*10*). Nonetheless, subsequent data demonstrate that S-protein-directed antibodies can have significant efficacy and safety, and both the Lilly and Regeneron antibody cocktail candidates received Emergency Use Authorization (EUA) from the US FDA in November 2020, although the EUA for Bamlanivimab was later withdrawn and distribution of the Bamlanivimab/Etesevimab cocktail is now limited to areas where resistant variant frequency is below 5%. Another S-protein specific antibody, Sotrovimab, co-developed by Vir and GlaxoSmithKline, received an EUA in May 2021. As publicly reported, Vir is currently developing a second-generation antibody aimed for use as a combination with Sotrovimab. The study published by Regeneron demonstrates that both Regeneron 2-Ab cocktail and Vir’s VIR-7831 antibody generated escape mutants after seven and two passages in vitro, outlining a need for multiple neutralizing antibodies in a cocktail (*11*). Omicron (BA.1, BA.1.1, and BA.2), escapes Regeneron and Lilly’s antibody cocktails, which led to the FDA’s decision to limit the use of bamlanivimab and etesevimab cocktail and REGEN-COV (casirivimab and imdevimab cocktail) only to patients infected with susceptible variants (that are currently not detected in the US) (*12*, *13*). The emergence of Omicron variants, believed to be masters of immune evasion (*14*), led the FDA to revise the EUA issued for another combination Evusheld (consists of tixagevimab and cilgavimab), and increased the dose due to loss of potency to BA.1 and BA.1.1 (*15*). Finally, a new Lilly’s antibody bebtelovimab, that received an EUA in February of 2022, retains some activity against Omicron (*16*). However, earlier findings from monoantibody therapies confirm the need for a cocktail treatment to avoid generation of escape mutants. Therefore, an antibody cocktail with broad reactivity and limited possibility of escape to current and prospective VOC that consists of several antibodies to block the generation of escape mutants is an urgent, yet unmet, medical need.

Small molecule inhibitors (SMI), which target viral proteins other than S protein, are alternatives to antibody-based therapies that might not be affected by the current VOC. However, SMI have additional limitations, such as the requirement to inhibit patient’s CYP3A for a viral protease inhibitor PF-07321332 or a low enough dose to avoid the host DNA mutagenesis for a ribonucleoside analog molnupiravir (*17*). In addition, SMIs are associated with toxicity concerns that could limit clinical usefulness for some patient populations, such as immunocompromised individuals (*18*, *19*). While paxlovid (PF-07321332 plus CYP3A inhibitor) reduced the risk of hospitalization in a selected group of COVID-19 patients, recent reports of a relapse of symptoms after a 5-day treatment led to a commentary from the FDA about the lack of evidence that an extended treatment provides any clinical benefit (reviewed in (*20*)). Thus, the collateral effects, potential consequences for immunocompromised patients and resistance patterns of SMI may need to be considered prior to patient dosing.

We previously reported the identification of a library of patient-derived antiviral antibodies using Immunome’s Discovery Platform (*21*). Based on published reports (*22*), we hypothesized that a cocktail of rare patient-derived immunoglobulins specific to conserved non-overlapping epitopes on S protein would have a synergistic antiviral effect and be resistant to mutational drift. In this report, we describe a cocktail of three patient-derived antibodies, IMM-BCP-01, that potently neutralized Omicron variant in a hamster model of SARS-CoV-2 infection in vivo, had a broad neutralization profile against Alpha, Beta, Gamma, Delta, Epsilon, Kappa, Lambda, Mu, Zeta and Omicron BA.1 and BA.2 variants in vitro*,* and caused reorganization and dissociation of Spike Trimer protein upon binding. Our experiments show that IMM-BCP-01 is a promising candidate to combat emerging SARS-CoV-2 variants infection in patients.

## RESULTS

### IMM-BCP-01, a combination of IMM20184, IMM20190 and IMM20253 antibodies, bound to conserved non-overlapping epitopes of Spike Trimer leading to its re-organization and dissociation into S monomers

**Fig. 1.**
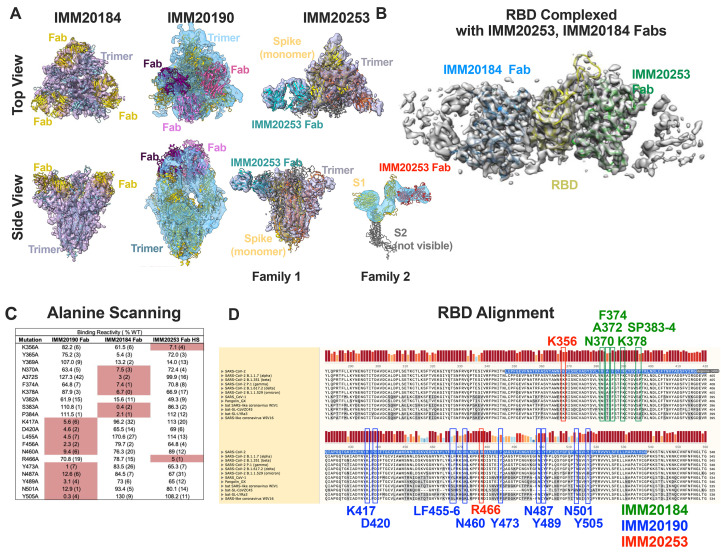
IMM20184, IMM190 and IMM20253 antibodies bind to conserved epitopes and disrupt Spike Trimer. (A) 3D reconstruction of Cryo-EM images of the Spike Trimer complex with either IMM20184 (left), IMM20190 (middle) or IMM20253 (right) Fabs. Top-to bottom view (top) and side view (bottom) are shown. IMM20253 Fab binding to Trimer results in two families, Family 1 and 2. Models PDB:7E8C, PDB:6XLU, PDB:6XM5 or PDB:7NOH were fit to density in Chimera for the Spike Trimer and PDB:6TCQ for the Fabs (see Supp Fig. 1A-E for details). (B) 3D reconstruction of Cryo-EM images of RBD complexed with simultaneously bound Fabs of IMM20184 and IMM20253. Fab model PDB:1M71 was fit to density in Chimera (see Sup Fig. 1F-J for details. (C) Critical residues of antibody epitopes identified as binding pattern to a library of single-point RBD mutants expressed on the cell surface. (D) Alignment of Spike protein sequences from current and prior CDC VOCs, SARS-CoV-1 and closely related coronaviruses. Critical residues of IMM20190, IMM20184 and IMM20253 epitopes are shown in blue, green and red. Highlighted sequence indicates RBD.

**Fig. 2.**
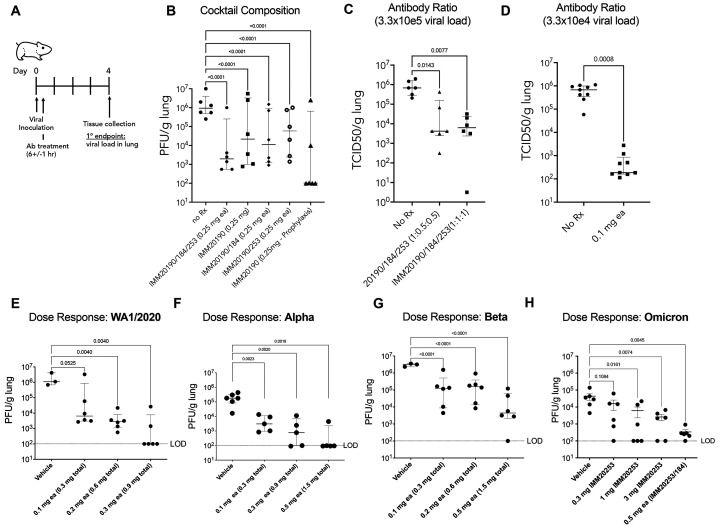
Three antibody combinations IMM20190/184/253 inhibits replication of non-adapted SARS-CoV-2 in lungs of infected animals. (A) All studies were carried out in Syrian Golden hamsters challenged with an intra-nasal inoculation of SARS-CoV-2 and treated with antibodies post-inoculation. Lungs were harvested at Day 4 and viral titers determined by either plaque forming or TCID50 assays. (B) Animals were infected with WA_CDC-WA1/2020 isolate and treated with single, double, or triple antibody cocktails, as noted, 6 hours post-inoculation. (C) Hamsters were challenged with 3.3 × 10^5^ TCID50 viral inoculation and treated with the three-antibody cocktail, at two different antibody ratios (1:1:1 or 1:0.5:0.5). (D) Hamsters were challenged with 3.3 × 10^4^ TCID50 viral inoculation and treated with the three-antibody cocktail at 1:1:1 ratio, at 0.1 mg dose each (0.3 mg total). (E -H) Hamsters were challenged with 10e4 PFU of WA1/2020 (E), Alpha (B.1.1.7) (F) Beta (B.1.351) (G), or Omicron (BA.1) (H) SARS-CoV-2 isolates after pre-treatment (Day -1) with different doses of IMM20253, IMM20184/253 or the three-antibody cocktail IMM20190/184/253 at equimolar ratios. Bar denotes median values. Error bars denote interquartile range. Statistical analysis in panel B is Two-Way ANOVA and in panels C-H is One-way ANOVA using Dunnet’s multiple comparisons test comparing to untreated group (No Rx).

**Fig. 3.**
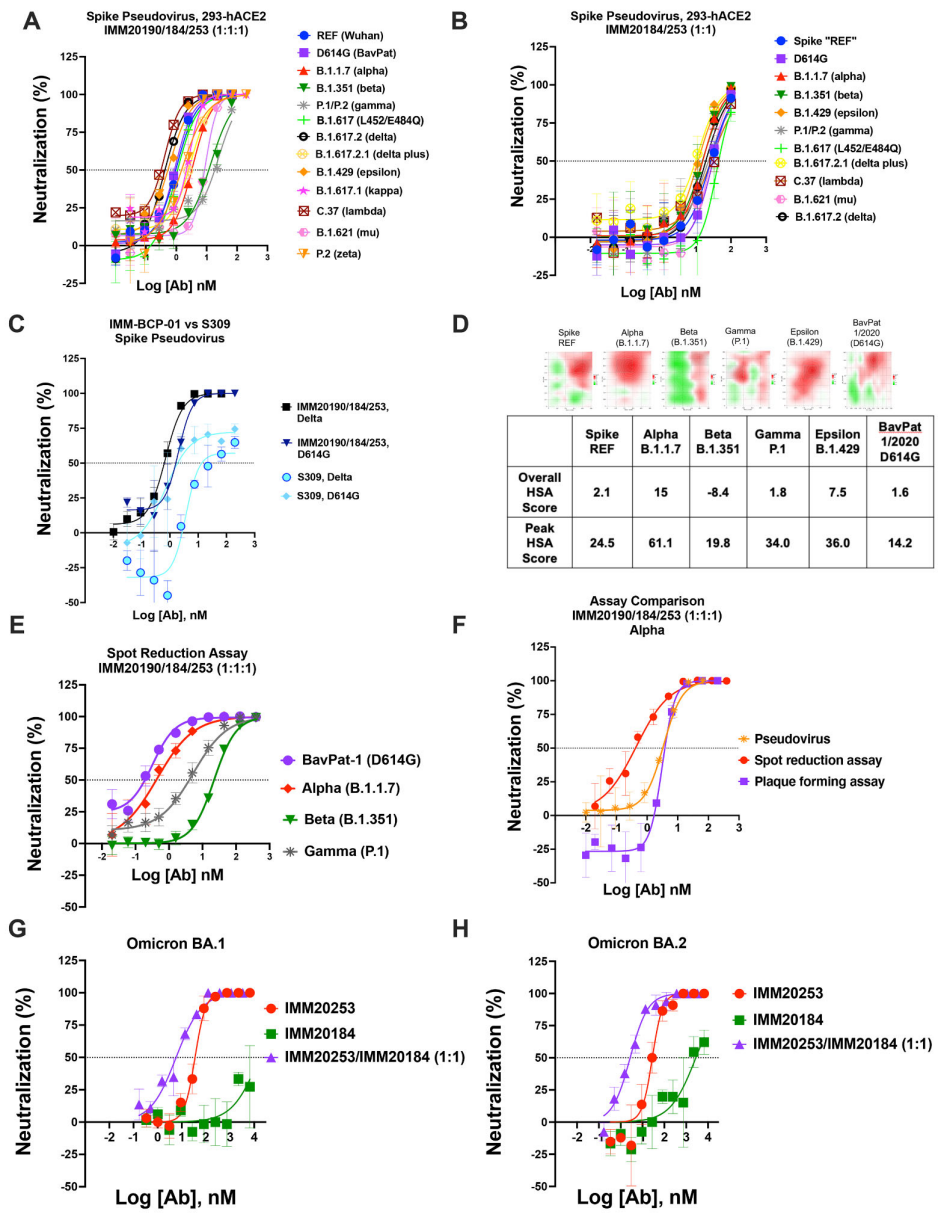
Immunome antibody combination has a combinatorial effect against clinically relevant SARS-CoV-2 variants. (A) Neutralization of Spike pseudoviruses that correspond to CDC VBMs and VOC in the presence of IMM20190/184/253 and (B) IMM20185/253 combination. Denoted points are Means of four replicates. Error bars denote SD. (C) Neutralization of D614G and Delta Spike pseudoviruses by the IMM-BCP-01 cocktail and S309 antibodies. Shown data are representative experiments of two independent repeats. (D) Synergy heatmaps (top) and scores (bottom) of the IMM-BCP-01 cocktail against five pseudoviruses and one BavPat1/2020 (D614G) live virus isolate calculated with SynergyFinder 2.0 online tool. HSA, the highest single agent model score, calculates the excess over the maximum single antibody response. Heatmaps (top) depict concentration-dependent HSA distribution, negative HSA in red and positive HSA in green. HSA score (bottom) below -10 indicates competition; between -10 to 10 shows additive effect; and above 10 demonstrates synergy among tested agents. (E) Neutralization of authentic VBM isolates of SARS-CoV-2 by the IMM-BCP-01 cocktail as measured in ViroSpot assay. Antibodies are mixed at equimolar ratio. Denoted points are Means of four replicates. Error bars denote SD. (F) Comparison of potency of the IMM-BCP-01 cocktail against the Alpha variant measured by three different methods, including a pseudovirus neutralization, intact virus spot reduction (ViroSpot) and intact virus plaque formation assays. (G) Neutralization of Omicron BA.1 and Omicron BA.2 (H) virus isolates by IMM20184, IMM20253 and IMM20184/253 combination as measured by plaque reduction assay. Denoted points are Means of four replicates. Error bars denote SD.

**Fig. 4.**
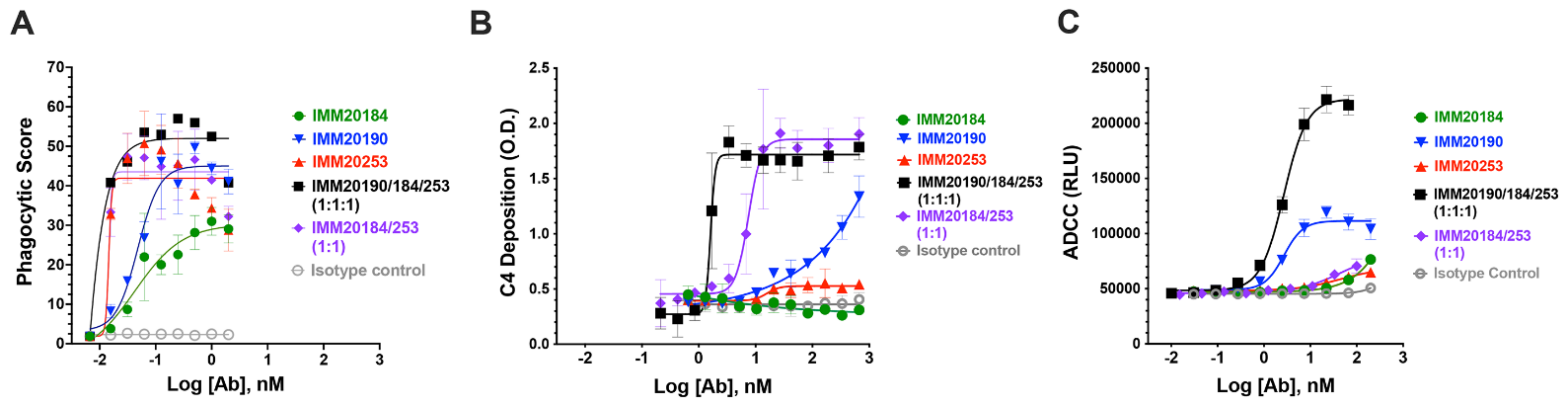
**Three antibody cocktail activates potent effector function responses in vitro*.*** (A) Phagocytosis of Trimer-coated beads opsonized with single IMM antibodies and two antibody (IMM20184/20253) and three-antibody (IMM190/184/253) combinations . Denoted points are means of three replicates. Error bars denote SD. (B) Deposition of classical complement component C4 on IMM antibodies bound to Trimer-coated surface. Denoted points are means of four replicates. Error bars denote SD. (C) Activation of antibody-mediated cellular cytotoxicity (ADCC) by IMM antibodies and two antibody (IMM20184/20253) and three-antibody (IMM190/184/253) combinations bound to S-expressing cells. Denoted points are mean; error bars are SEM.

Using an unbiased interrogation of a previously described library of patient-derived antiviral antibodies (*21*), we identified three monoclonal antibodies (mAbs), IMM20190, IMM20184 and IMM20253, that had robust additive and synergistic combinatorial antiviral effects. Structural (**Fig. 1** **and Supp** **Fig. 1**) and functional (**Fig. 2**, **Fig. 3**, and **Fig. 4**) studies of these antibodies revealed trimer reorganization and subsequent dissociation into Spike monomers upon IMM20184 and IMM20253 binding (**Fig. 1** **and** **5**).

**Fig. 5.**
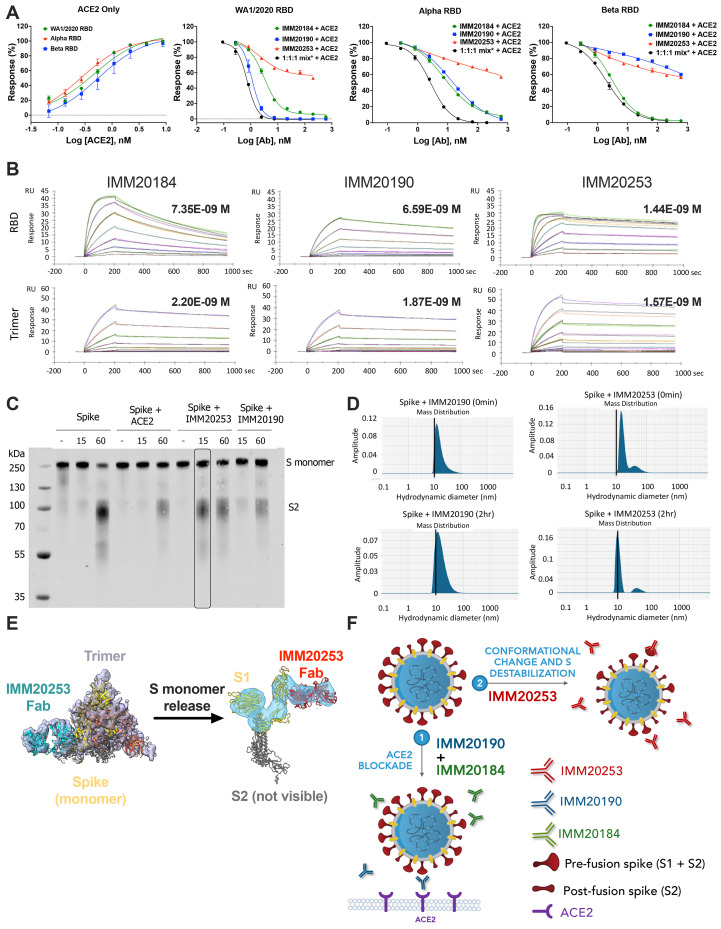
IMM20253 antibody inhibits virus in non-ACE2 dependent manner and facilitates the release of S1 protein. (A) Inhibition of RBD binding to its cellular receptor ACE2 in the presence of IMM20184/190/253. ELISA-based receptor competition assay. Denoted points are means of three replicates. Error bars denote SD. (B) Antibody binding kinetics of IMM20184, IMM20190 and IMM20253 antibodies to soluble RBD and Trimer (WA1/2020 variant) measured using Surface Plasmon Resonance (SPR). Denoted values are KD. (C). Western blot analysis of Trimer digested with protease K after 0, 15 and 60 min in the presence of either human ACE2, IMM20253 or IMM20190. Anti-S2 staining reveals S monomer (S1+S2) and S2 protein. (D) Dynamic light scattering (DLS) analysis of Trimer complex with IMM20253 or IMM20190 immediately or after 2 hours incubation measures a hydrodynamic diameter of each complex in nm. (E) IMM20253 Fab binding to Trimer triggers complex disruption and release of S monomers. (F) Schematic of mechanism of action of the IMM-BCP-01 cocktail.

Structural analysis of an S protein trimer (Trimer) complexed with bound Fabs of IMM20184, IMM20190 or IMM20253 (**Fig. 1A**) identified binding patterns of the IMM-BCP-01 antibodies. A final 3D reconstruction of cryo-electron microscopy (cryo-EM) micrographs of IMM20184 Fabs bound to Trimer reveals a 3:1 (Fab:Trimer) complex at ~8.3-10 Å resolution (Supp Fig. 1A, D) with a decreased density in the Trimer core indicating protein rearrangements (**Fig. 1A** **and Supp. Figure 1A,D**). Cryo-EM micrographs of IMM20190 Fab complexed with Trimer revealed a 3:1 (Fab:Trimer) complex at ~6.9-7.9 Å (**Fig. 1A** **, Supp Fig. 1B, D)**. While the variable regions of IMM20190 Fabs was clearly resolved, the constant regions were scattered, suggesting a dynamic binding nature of this antibody. Finally, the cryo-EM analysis of IMM20253 Fab-Trimer complex was repeated twice with 3:1 and 6:1 molar ratios (Fab:Trimer), with the same unexpected conclusion. The samples were not aggregated, observed with good contrast, and clearly converged into two structural families (**Fig. 1A** **and Supp** **Fig. 2C, D)**. The first family consisted of 1 Fab:1 Trimer complex that has one S monomer partially unfolded (as revealed by a lower density). The second family includes smaller complexes that converge into a 3D structure of IMM20253 Fab bound to S1 (**Fig. 1A**, *side view*, and **Supp** **Fig. 1C, D)**. The S2 portion of the spike monomer was not visible in the density maps, suggesting that it moved freely in the complex relative to the S1 domain.

The Trimer reorganization induced by both IMM20184 and IMM20253 Fabs prompted us to determine their binding pattern at higher resolution. Cryo-EM structures of RBD with simultaneously bound Fabs of both IMM20184 and IMM20253 were resolved to ~4.0-4.6 Å (**Fig. 1B** **, Supp.** **Figure 1F-J**). To achieve this resolution, The final reconstruction included ~3 × 10^5^ particles and produced a map at ~4 Å nominal resolution (**Fig. 1B** and **Supp. Figure 1G-J**). The complex structure demonstrated that both IMM20184 and IMM20253 Fabs simultaneously bound to RBD protein. Consistent with **Fig. 1A**, the epitope of IMM20253 was located on the outer surface of the RBD, whereas the epitope of IMM20184 faced inward and sideways, potentially enabling avid binding of IMM20184. Of note, binning of IMM20184/190/253 antibodies using bio-layer interferometry (BLI) confirmed the cryo-EM data and showed that the antibodies did not compete for binding of S (**Supp.** **Figure 1K**).

The structural data was further confirmed through use of an alanine-scanning shotgun mutagenesis approach (*22*) (**Fig. 1C**). In brief, we used a validated library of RBD (Wuhan) proteins expressed on the surface of HEK-293T cells, each containing one amino acid mutation (*22*). Consistent with cryo-EM data (**Fig. 1A,B** **and Supp.** **Figure 1**), mutagenesis identified rare non-overlapping epitopes for the three antibodies (**Fig. 1C**). IMM20184 bound to a highly conserved region in the core RBD (**Fig. 1**). The binding site laid in close proximity to the previously reported epitopes of CR3022 and COVA1-16 antibodies that bind to a cryptic epitope on RBD (*23*, *24*). The IMM20184 epitope included residues N370, F374, K378, and SP383-384 that were completely conserved among all current and previous SARS-CoV-2 VOC (**Fig. 1D**), including Omicron BA.1 and BA.2 variants.

IMM20190 bound to an epitope that included the receptor-binding ridge and an area adjacent to the receptor-binding loop. The epitope mapping analysis identified 10 residues in the RBD that interact with IMM20190. Of these, two residues, K417 and N501 were mutated, either singly (K417 in Alpha/B.1.1.7) or doubly (K417/N501 in Beta, Gamma, or Omicron) in prior and present VOC. The Delta variant is conserved at all 10 interaction residues (**Fig. 1D**)s. The broad epitope may explain the resistance of IMM20190 to the majority of single- and double-point mutations within the RBD region (**Supp.** **Table 1**) and the flexible nature of Fab binding observed by Cryo-EM (**Fig. 1A**).

Alanine scanning mutagenesis identified only two critical residues for IMM20253 binding to RBD, K356 and R466, located on the outer surface of the RBD. This complements the cryo-EM data (**Fig. 1A, B**). K356 resides within the surface area that interacts with the VL, and R466 resides within the surface area that interacts with the VH of IMM20253. R466 residue was conserved in all sarbecoviruses or lineage b betacoronaviruses, whereas K356 was conserved in most (**Fig. 1D**) and (*23*, *25*, *26*)). In summary, IMM20253 bound to a highly conserved epitope on the outer surface of RBD, does not compete with IMM20184 and IMM20190 for RBD binding and induces dissociation of Trimer complex into monomers.

### The IMM-BCP-01 cocktail suppressed the severity of the disease in an in vivo model of SARS-CoV-2 infection

We tested the efficacy of different combinations and doses of IMM20184, IMM20190 and IMM20253 in Syrian Golden hamsters inoculated with SARS-CoV-2 (WA_CDC-WA1/2020) (**Fig. 2A**). When administered to animals 6 hours after viral challenge (treatment paradigm), we observed that IMM20190 or 2-Ab combinations of IMM20184/IMM20190 or IMM20190/IMM20253 led to robust viral clearance in the lungs (**Fig. 2B**). However, the greatest clearance was observed with the 3-Ab cocktail. Five of the six animals in this cohort showed an approximately 2.5-log10 reduction of viral titer in the lung on day 4 post viral challenge. In a follow-up study, the 3-Ab cocktail decreased the viral titer in the lungs of animals inoculated with a high-titer (3.3 × 10^5^ TCID50) of WA_CDC-WA1/2020 (REF variant) by over 100-fold. This efficacy was observed when the antibodies were administered at either 1:1:1 (p=0.0077) or 1:0.5:0.5 (IMM20190:IMM20184:IMM20253; p=0.0143) molar ratios (**Fig. 2C**). However, when subjected to an F-test, the variability in clearance level in 1:0.5:0.5 group was higher (P < 0.0001) than when animals were treated with an equimolar ratio cocktail (**Fig. 2C**). These studies were performed using a viral inoculum that was approximately 10-fold higher than what is typically used to evaluate efficacy of antibody therapies (*25*, *27*). When repeated at a lower inoculating dose (3.3 × 10^4^ TCID50 of WA1/2020 variant per animal) (**Fig. 2D**), treatment of hamsters with the IMM-BCP-01 (0.1 mg each, 0.3 mg total antibody) resulted in a significant (p<0.0080) ~3.5 log10 decrease in viral titer relative to vehicle-treated controls. Taken together, data in this experiment support the IMM-BCP-01 cocktail as comprising all three antibodies at 1:1:1 ratios to obtain the most consistent level of viral clearance.

### IMM20184/190/253 antibodies and their combinations dose-dependently inhibit virus load in lungs of hamsters infected with WA1/2020, Alpha, Beta and Omicron variants.

The IMM-BCP-01 cocktail was designed to recognize and inhibit variants that have and could emerge. Consistent with that goal, the IMM-BCP-01 exhibited a dose-dependent inhibition of all viral variants tested in vivo, including the reference (WA1/2020) variant, Alpha, Beta and Omicron BA.1 isolates (**Fig. 2E-H**). The three-antibody cocktail suppressed viral infection in the lungs of hamsters pre-treated with doses as low as 0.1 mg of each antibody (0.3 mg total dose) 24 hours prior to virus challenge. Higher doses of the IMM-BCP-01 lowered viral loads in hamsters to a greater degree. A 10,000-fold reduction in viral load in lungs of animals inoculated with WA1/2020 (**Fig. 2E**), and a 1,000-fold reduction in animals inoculated with Alpha **(** **Fig. 2F** **)** and Beta **(** **Fig. 2G** **)** isolates were achieved with doses of 0.3 and 0.5 mg each (0.9 mg and 1.5 mg total for a IMM20190/184/253 cocktail). Animals infected with Omicron BA.1 isolate **(** **Fig. 2H** **)** developed a lower viral lung load (~4.4*10E4 PFU/g) comparing to other isolates, that was dose-dependently reduced by a standalone IMM20253 antibody. The two-antibody combination IMM20153/IMM20184 (0.5 mg ea or 1 mg total dose) further decreased viral load in lungs to levels comparable to the lower limit of detection (LOD) for the study **(** **Fig. 2H** **)**. Thus, IMM20184, IMM20190 and IMM20253 antibody combinations potently suppresses infection of multiple SARS-CoV-2 variants in vivo in a dose-dependent manner.

### IMM-BCP-01 cocktail pharmacokinetics profile and exposure

To evaluate the cocktail pharmacokinetics profile, naïve hamsters were dosed with IMM-BCP-01 via a single intraperitoneal injection. Concentration of total human IgG (combination of three antibodies) in serum was measured at different time points after dosing by ELISA. The IMM-BCP-01 cocktail generally followed first-order absorption and elimination process with a half-life of approximately 100 hours in hamsters (**Supp** **Fig. 2A, B**). To study the dependence of viral titers in lungs of WA1/2020 SARS-CoV-2 infected hamsters on the concentration of IMM-BCP-01, we tracked terminal exposures of total human IgG in blood (**Sup** **Fig. 2C**). Those studies demonstrated that variability in viral clearance correlated directly with systemic distribution of IMM-BCP-01 (**Supp** **Fig. 2C**). Animals that exhibited lower viral lung titers were associated with terminal plasma levels of the IMM-BCP-01 greater than 3-5 μg/mL. In contrast, the IMM-BCP-01 was not observed at appreciable levels in the blood of animals that failed to clear virus from the lungs, that rather reflected the difficulties with antibody injection to these animals. Effective levels of IgG in the blood were achieved with dose levels as low as 0.1 mg each (0.3 mg total dose) in treatment settings when the drug was absorbed and systemic exposure was achieved (**Suppl. Figure 2C**).

### IMM-BCP-01 had a combinatorial neutralizing effect against SARS-CoV-2 VOCs

The IMM-BCP-01 cocktail was evaluated in three live (authentic) virus neutralization assays and one reporter pseudovirus assay (**Fig. 3** **and Sup** **Fig. 4**) using an array of viral variants. The three independent authentic virus neutralization assays provided comparable data (**Tables 1** **,** **2**), which agreed with pseudovirus neutralization results (**Table 3**). The antibody cocktail neutralized all tested variants being monitored (VBM) and variants of concern (VOC) (**Fig. 3**). The IMM-BCP-01 cocktail (IMM20184/190/253), as well as IMM20184/20253 combination, completely neutralized all pseudovirus variants tested (**Fig. 3A,B**). Overall, IMM-BCP-01 potently neutralized the spectrum of variants tested, with all IC50 values being within 2-log of the reference pseudovirus encoding a WA1/2020 S protein. The three-antibody cocktail had a modest, but reproducible, *increase* in potency against Delta, Lambda (C. 37), and Epsilon (B.1.429) pseudoviruses, which could be explained by a higher susceptibility of Trimers from these variants to structural rearrangements. In context of current landscape of antibody therapeutics for COVID-19, IMM-BCP-01 outperformed S309 against Delta and a WA1/2020 D614G pseudovirus (**Fig. 3C** **)**. S309 is the parental clone of VIR-7831, which obtained an EUA and retains activity against some Omicron variants (*27*).

**Table 1.** Neutralization potency of IMM20184, IMM20190 and IMM20253 antibodies and their combinations against four virus isolates in a spot reduction assay. Denoted IC_50_ (nM) values are means of four replicates .

**Antibody**	**BavPat (D614G)**	**Alpha**	**Beta**	**Gamma**
IMM20184	33.8	43.3	81	18.4
IMM20190	0.4	2.7	>393	>393
IMM20253	39.4	1.4	155.4	13.4
IMM20184/IMM20190	0.4	1	Not Tested	
IMM20184/IMM20253	3.9	5.7		
IMM20190/IMM20253	0.4	0.9		
IMM-BCP-01	0.24	0.4	21.2	4.2

**Table 2.** Neutralization potency of IMM20184, IMM20253 antibodies and IMM20184/253 antibody combination against Omicron BA.1 and BA.2 virus isolates in a plaque reduction assay. Denoted IC_50_ (nM) values for WA1/2020 and Omicron BA.1 are Means from two and three independent experiments. Denoted IC_50_ (nM) values for BA.2 are means of three replicates from a single experiment.

**Antibody**	**WA1/2020** (N=2)	Omicron BA.1 **(N=3)**	Omicron BA.2 **(N=1)**
IMM20184	81.5	>1000	>1000
IMM20253	27.3	48.2	28.3
IMM20184/IMM20253	19.0	16.7	2.7

**Table 3.** Neutralization potency of the IMM-BCP-01 cocktail (IMM20184/190/253 antibody combination) against various pseudovirus Spike variants. Denoted IC_50_ (nM) values are means of four replicates.

**Isolate**	**IC_50_ (nM)**
REF (WA1/2020)	1.0
BavPat (D614G)	0.6
Alpha	3.0
Beta	13.5
Gamma	24.8
B.1.617 (L452/E484Q)	1.0
Delta	0.4
Delta plus	3.0
Epsilon	0.6
Kappa (complete sequence)	2.7
Lambda	0.4
Mu	9.1
Zeta	1.53

Abbreviations: IC_50_ = half maximal inhibitory concentration; REF = reference.

To better understand the combinatorial activity of IMM20184, IMM20190 and IMM20253 antibodies, we performed a series of experiments focusing on the contributions of each antibody to the overall neutralizing activity (**Supp** **Fig. 3,** **Fig. 3D**). We tested the two-antibody mixtures of IMM20190 (1x concentration) with either IMM20184 (1x) or IMM20253 (1x), and a three-antibody combination of IMM20190 (1x) with IMM20184/IMM202053 (0.5x each) in pseudovirus neutralization assays (**Supp. Figure 3**). Two- and three- antibody combinations dose-dependently neutralized pseudovirus variants corresponding to WA1/202, Alpha, Beta, Epsilon and Gamma (**Supp. Figure 3A, B)**. We calculated each antibody contribution to the observed neutralizing effect using SynergyFinder 2.0 (*28*). A score below -10 suggests an antagonistic (competitive) effect; a score between -10 and 10 reflects an additive effect; and a score above 10 suggests a synergistic effect of the combined treatment. We detected a concentration-dependent synergistic potential of combinations (**Supp.** **Figure 3C**). In variants that IMM20190 potently neutralized, such as WA1/2020 and Epsilon (**Supp Fig. 3A**), antibody combinations are mainly additive, as IMM20190 neutralization was sufficient and did not require the two other antibodies. In variants where the potency of IMM20190 was reduced, such as Beta and Gamma, combinations were also additive. In addition, IMM20184 and IMM20253 antibodies as a double combination had an additive neutralizing effect against these variants (**Supp.** **Figure 3D**). We observed the highest synergistic potential of the three-antibody combination IMM20190/184/253 for the Alpha variant, where each of the single antibodies neutralized with comparable IC_50_’s **(** **Fig. 2D** **)**. In the Alpha variant, the three-antibody combination outperformed all three of the individual antibodies. Thus, the three-antibody cocktail neutralized all tested variants and was associated with additive or synergistic effects depending on the strain. Combined with the observed antibody pharmacokinetics (**Supp.** **Figure 2A**), these data suggest that administration of the three-antibody cocktail (0.5 mg each) reaches serum concentrations in vast excess of the IC50 neutralization concentrations observed for all SARS-CoV-2 variants tested (**Supp. Figure 2, and** **Fig. 2** **,** **3**).

We evaluated the potency of the IMM20184/190/253 antibodies and their combinations against current and prior variants of concern in the authentic virus neutralization assays. IMM-BCP-01 neutralization of WA1/2020, BavPat (D614G), Alpha, Beta, and Gamma variants was measured in a spot reduction assays (**Fig. 3E** **)** and IMM20184/253 neutralization of Omicron BA.1, BA.1.1 and BA.2 in a front reduction and plaque reduction assays (**Fig. 3G,H** **and Sup.** **Figure 4**). For certain viral isolates, such as Alpha variant **(** **Fig. 3F** **and** **Tables 1** **-** **3** **)** we observed equivalent or better potency of the IMM-BCP-01 cocktail as compared to the corresponding pseudovirus neutralization assay (**Fig. 3F** **and** **Tables 1** **-** **3**). Consistently with the data observed in vivo (**Fig. 2H**), a standalone IMM20253 antibody neutralized Omicron BA.1 and BA.2 variants in a plaque reduction neutralization assay (**Fig. 3G, H**) and BA.1.1, harboring an additional R346K mutation in a focus reduction neutralization assay (**Supp.** **Figure 4**). Although no mutations present in the Omicron isolates mapped to critical binding residues for IMM20184 (**Fig. 1C**), the standalone IMM20184 antibody did not reach a complete neutralization of Omicron BA.1 and BA.2 (**Fig. 3G, H**). A complete loss of neutralizing activity of IMM20184 alone was observed against BA.1.1 in the context of the FRNT assay (**Supp.** **Figure 4**). The IMM20184/253 combination showed a combinatorial effect against Omicron BA.1 and BA.2 isolates, compared to the IMM20253 antibody alone, in the plaque reduction assay (**Table 2**) and (**Fig. 3G, H**), which was in agreement with the result observed using Omicron isolate in vivo (**Fig. 2H**).

Finally, we observed a higher in vivo potency of the IMM-BCP-01 cocktail compared to its activity by virus neutralization assays in vitro. We detected a 100-fold increase in EC50 of the IMM-BCP-01 cocktail against the Beta variant in both pseudovirus (**Fig. 3A**) and authentic virus (**Fig. 3E**) assays in vitro that only resulted in a minor dose increase (from 0.3 mg to 0.5 mg per antibody) in vivo (**Fig. 2F, G** **and** **3A**), outlining the importance of in vivo studies for anti-SARS-COV-2 antibodies. Thus, IMM20184/190/253 antibodies and their combinations potently neutralized all tested SARS-CoV-2 variants, including Omicron BA.1 and BA.2, in vitro.

### IMM20190/184/253 antibody cocktail activated potent effector responses in vitro*.*

A growing body of evidence suggests that intact effector functions are required for optimal viral clearance in animal models of COVID-19 (*29*–*31*). The antibodies comprising the IMM-BCP-01 cocktail retain intact IgG1 Fc domains and bind to the RBD in a non-competitive manner (**Fig. 1** **, Supp.** **Figure 1H**). We thus hypothesized that IMM2019/184/253 might generate an oligoclonal response to S protein that activates Fc-mediated effector functions including antibody-dependent cellular cytotoxicity (ADCC), antibody-dependent cellular phagocytosis (ADCP), and classical complement pathway (CP) **(** **Fig. 4** **)**. To test this hypothesis, we first measured antibody-induced phagocytosis of Trimer-coated beads using a published method (*32*). All three human antibodies induced phagocytosis of Trimer-coated beads in a dose-dependent manner relative to an IgG1 isotype control (**Fig. 4A**). Even a low (~15 pM) concentration of IMM20253 antibody potently induced phagocytosis. The three- antibody cocktail (IMM20190/184/253) demonstrated a higher phagocytic score than a two-antibody cocktail (IMM20184/253) or each individual antibody (**Fig. 3A**). We did not observe phagocytosis of Trimer-coated beads in the presence of an IgG1 isotype control antibody. Next, we evaluated activation of the classical CP by IMM20190/184/253 cocktail (Fig. 3B). In brief, we adapted a CP activation assay (*33*) and measured deposition of the complement component C4 from serum of normal human donors on anti-S antibodies bound to Trimer-coated surface. IMM20190 and IMM20253 antibodies bound to Trimer promoted detectable levels of C4 deposition. While IMM20184 binding to Trimer alone did not activate CP in this assay, the two-antibody cocktail IMM20184/253 induced C4 deposition on antibody-Trimer complexes (**Fig. 3B**). The three antibody cocktail IMM20190/184/253 induced the most robust activation of C4 deposition. Since all tested antibodies had the same intact heavy chain IgG1 Fc region, we hypothesized that C4 deposition on Trimer-antibody(ies) immune complex might depend on the Fc epitope conformation and accessibility as previously demonstrated for other antibodies (*34*, *35*). Cryo-EM studies (**Fig. 1** **, Supp.** **Figure 1**) indeed demonstrated that the IMM-BCP-01 cocktail attacks Trimer from different directions and may indeed create an array of Fc regions that facilitates binding of C1q. Finally, ADCC assays revealed a similar combinatorial effect among IMM20184/190/253 antibodies (Fig. 3C). While each antibody induced a mild (IMM20184, IMM20253) to moderate (IMM20190) activation of ADCC, the three antibody cocktail IMM20190/184/253 induced the greatest response (**Fig. 3C**). Therefore, IMM20184/190/253 antibodies and their combinations activated a potent combinatorial effector response.

### IMM20184, IMM20190, but not IMM20253, blocked S interactions with ACE2

The location of IMM20184 and IMM20190 epitopes suggests that these two antibodies likely block ACE2 binding. To test this hypothesis, we performed a biochemical ELISA-based receptor inhibition assay. The affinity of a soluble ACE2 protein to Wuhan-1, Alpha, and Beta variant RBDs coated to an ELISA plate was evaluated in the presence of various concentrations of antibodies of interest. Consistent with the data from the homogeneous time resolved fluorescence (hTRF) assay (**Supp.** **Table 1**), IMM20184 potently inhibited ACE2 binding to all three RBD variants (**Fig. 5A**). IMM20190 blocked ACE2 binding to Wuhan-1 and Alpha variant RBD proteins, and partially decreased ACE2 binding to the Beta variant RBD. In contrast, IMM20253 did not fully block ACE2 interactions with any of the three RBD variants tested (**Fig. 5A**). A partial inhibition of ACE2 binding by IMM20253 (up to 40%, depending on the concentration) was detected for all three S variants tested. These data were consistent with the location of the IMM20253 epitope relative to the ACE2 binding site and suggest its neutralization occurred through a distinct mechanism of action. Finally, an equimolar mixture of IMM20190, IMM20185, and IMM20253 antibodies disrupted ACE2 binding to all three tested RBD variants. The inhibitory effect of a three-antibody cocktail was more pronounced than the effect of each individual antibody. Of note, we detected a minor ACE2 binding preference to Alpha RBD than to Beta RBD variant (**Fig. 5A**, *left panel*). Thus, IMM20253 partially, and IMM20184 and IMM20190 completely blocked RBD interaction with its cellular receptor ACE2.

### IMM20190, IMM20184 and IMM20253 bound to soluble RBD and S1 proteins recapitulating different SARS-CoV-2 variants

Steady-state binding of the three antibodies across a range of variants was characterized via hTRF (**Supp.** **Table 1** **and Supp.** **Figure 5**). Each of the three antibodies was tested for binding against isolated RBD or S1 protein encompassing over 20 different single and multiple mutations that correspond to naturally occurring and predicted escape mutations. IMM20184 and IMM20253 retained picomolar EC_50_ binding to most of single and multiple point mutations tested, including those present in VBM and VOC. Furthermore, IMM20253 exhibited some binding to SARS-CoV-1 Spike. In contrast, IMM20190 binding was reduced, to varying degrees, by K417N and the series of RBD-localized mutations associated with K417N/E484K/N501Y or K417T/E484K/N501Y variants. Binding of IMM20190 appeared to be partially restored for two different S variants containing D614G even in the presence of K417N (**Supp. Table 1**). Thus, the IMM-BCP-01 cocktail demonstrated binding by at least two antibodies for each tested SARS-CoV-2 variant.

### Analysis of binding kinetics of IMM20184, IMM20190, and IMM20253

Antibody binding kinetics were measured using a multi-cycle kinetics protocol on a Biacore T200 surface plasmon resonance (SPR) instrument. While all three antibodies bound with high affinity to both RBD and Trimer Spike proteins. (**Fig. 5B**), antibody binding to RBD, measured as on-rates (k_on_), happened faster (i.e., k_on_ rates were higher) (**Table 4**). This effect was least pronounced for IMM20190, suggesting that its epitope was the most accessible in the intact Trimer structure. IMM20184 and IMM20253 bound to the RBD 17- and 19-fold faster than to the Trimer, respectively. The dissociation of IMM20184 from Trimer (k_off_^Trimer^ = 2.3E-04 1/s) was 6-fold slower than from RBD (k_off_^RBD^ =1.3E-03 1/s). This difference in dissociation suggests the antibody/Trimer complex is stabilized through an avid binding mechanism between the antibody and two subunits of the Trimer, consistent with the epitope mapping data (**Fig. 1**). IMM20253, despite exhibiting the greatest difference (19-fold) in on-rates between the RBD and Trimer, has the fastest on-rate for the Trimer of all three antibodies. This result suggests the IMM20253 epitope is readily accessible in the context of the Trimer structure. Therefore, SPR data suggest that IMM20184 has an avid binding and IMM20253 has the highest affinity to Spike protein out of three tested antibodies.

**Table 4.** Affinity of IMM20184, IMM20190, IMM20253 antibodies to soluble RBD and Trimer Spike proteins. Kon, Koff, KD values measured on Biacore T200 using a multi-cycle kinetics protocol assuming 1:1 interaction model.

**Antibody**	**Analyte**	**k_on_(1/Ms)**	**k_off_(1/s)**	**kD (M)**
IMM20184	Trimer Spike	3.12E+04	2.29E-04	7.35E-09
	RBD	5.95E+05	1.31E-03	2.20E-09
IMM20190	Trimer Spike	2.95E+04	1.95E-04	6.59E-09
	RBD	2.02E+05	3.76E-04	1.87E-09
IMM20253	Trimer Spike	8.13E+04	1.17E-04	1.44E-09
	RBD	1.39E+06	2.18E-04	1.57E-10

### IMM20253 binding released Spike monomers and facilitated protease cleavage.

IMM20253 disruption of Trimer into Spike monomers, detected by cryo-EM (**Fig. 1**), suggested that the antibody might facilitate cleavage of S into S1 and S2. We used a previously published method to evaluate Trimer sensitivity to protease cleavage in the presence of an antibody (*36*). Briefly, Trimer protein was mixed with protease K in the presence of either (1) buffer only, (2) human recombinant ACE2 protein, (3) IMM20253 or (4) IMM20190, an anti-S antibody that recognizes ACE2-binding region, and incubated for 0, 15 and 60 min. Protease readily cleaved S incubated with buffer after 60 min. ACE2 or IMM20190 appeared to partially decrease protease cleavage at 60 min, perhaps due to steric hindrance. In contrast, IMM20253 induced S cleavage after 15 min (**Fig. 5C**). In a complementary experiment, we evaluated S samples preincubated with IMM20253 or IMM202190 in the absence of protease. Samples were analyzed using a standard “premix” protocol by dynamic light scattering (DLS). Incubation of S with IMM20190 led to the generation of complexes and increased the size of particles, measured as an increase in hydrodynamic diameter after 2 hours of incubation (**Fig. 5D**). Consistent with cryo-EM and protease cleavage results, incubation of S with IMM20253 *decreased* the hydrodynamic diameter of the resulting complexes, consistent with the complex disruption into monomers observed in Cryo-EM (**Fig. 1A** **and** **5E**). These data support a mechanism of action for the IMM20184/190/253 cocktail, whereby IMM20253 binding disrupts the Trimeric architecture of the S complex and facilitates cleavage into S1 and S2 in the presence of proteases, and IMM20190 and IMM20184 binding blocks the interaction with a cellular receptor ACE2 (**Fig. 5F**).

## DISCUSSION

We described three patient-derived antibodies that bind to non-competing epitopes on S protein, trigger Trimer reorganization into one resembling a post-fusion confirmation, and induce a potent antiviral response in vitro and in vivo. Particularly, we revealed that IMM20253 bound to a conserved epitope on Trimer Spike and induced complex dissociation into monomers and facilitated their cleavage into S1 and S2 subunits. When combined with IMM20190 and IMM20184, the three-antibody cocktail consistently showed robust antiviral potency in vivo and in vitro, neutralized all VOC and VBM tested, including Omicron BA.1 and Omicron BA.2, and induced a potent multicomponent effector response.

Each of the three antibodies comprising the IMM-BCP-01 cocktail appeared to elicit viral neutralization through different mechanisms. IMM20190 and IMM20184 antibodies competed with a cellular receptor hACE2 for binding to Spike protein. The IMM20190 epitope, identified by Cryo-EM and confirmed using mutagenesis, extended to two surfaces, including the receptor binding ridge of RBD and the region around the receptor-binding loop (*37*). Considering the breadth of this epitope, IMM20190 was shown to be resistant to small changes in the RBD sequence. The epitope of IMM20184 antibody is located in N370 – P384 region of the RBD and consists of 5 critical residues, surrounding the RBD core (*37*), that is conserved in Omicron and all prior VOC of SARS-CoV-2 and other SARS-related coronaviruses (*23*, *24*). IMM20184 antibody had a higher affinity to a soluble RBD, yet 5.7-fold slower dissociation from a soluble Trimer, indicating avid binding to Trimer. The COVA-1 antibody that binds to the area adjacent to IMM20184 epitope has strong cross-neutralizing properties due to its avid binding (*23*). The remaining antibody IMM20253 did not directly compete with hACE2, but bound to a conserved epitope (K356 and R466 residues) that is not a common target of the human immune system for generating neutralizing antibodies (*37*). IMM20253 binding led to a dissociation of Trimer into S protein monomers, that likely facilitated cleavage into S1 and S2 in the presence of proteases. Thus, IMM20253 triggered a conformational change of S protein into its post-fusion form and prevents binding to host cells in an ACE2-independent manner. The IMM20253 epitope is present in all human as well as in SARS-related coronaviruses (*26*) and is, therefore, expected to be retained in emerging human SARS-related viruses. K356 participates in a formation of a hydrophobic pocket in the RBD (*38*) that may explain its functional importance and evolutionary conservation. The conserved patch of amino acids around R466 has only rarely elicited an antibody response (*37*). R466 is conserved in all SARS-related pangolin and bat betacoronaviruses, making it an attractive target for the therapeutic intervention and vaccine design. There are two reported antibodies (a nanobody derived from llama and a mouse-derived antibody) that recognize larger patches of RBD and appear to have overlapping epitopes with IMM20253 (*36*, *39*). However, both described antibodies bound to broader epitopes, i.e more sensitive to mutational drift; both were generated via animal immunizations and need to be tested for off-target binding to human tissues; and either need to be humanized (mouse-derived) or re-designed (llama-derived) prior to consideration as therapeutics. These two studies, however, further confirm the importance of the IMM20253 epitope.

While Cryo-EM data confirmed the mechanism of action of IMM20184 and IMM20253 and were instrumental in identifying dissociated Spike monomers, there were limitations of this approach. Particularly, the original dataset exhibited a strong preferential orientation of Spike protein, that may lead to anisotropic reconstructions and artifacts in the density maps. This was especially important to overcome for a higher resolution structure of RBD complexed with IMM20184 and IMM20253 Fabs. Therefore, the original dataset needed to be appended by a tilted dataset, that used a stage tilt during data collection (*40*). The resulting 3D reconstruction still exhibited some degree of preferred orientation, but helped to resolve the binding sites of IMM20184 and IMM20253 Fabs at a higher resolution. Other approaches, such as overlaying cryo-EM grid with a thin layer of carbon or changing the formulation of the complex, could be used for subsequent studies to address the preferred orientation of spike protein in cryo-EM studies (*41*, *42*).

The range of neutralization potency exhibited by IMM-BCP-01, across the breadth of pseudovirus and live virus tested, translated into in vivo efficacy in animal models. This was illustrated most notably in the setting of the Beta and Omicron variants. Despite showing a variable level of in vitro neutralization potency (4.9 – 24 nM) against the Beta isolate depending on the assay used to measure activity, the IMM-BCP-01 cocktail exhibited robust in vivo efficacy at doses consistent with those currently being used in the clinic for other SARS-CoV-2 antibodies. While IMM-BCP-01 appeared to neutralize virus comparably to S309, the in vivo potency of IMM-BCP-01 exceeded that of VIR-7831 (*18*)*.* Consistent with the results obtained for Beta variant, a modest neutralization of Omicron variant by IMM20253 alone (49.5 nM) or by IMM20184/20253 combination (22 nM) in vitro translated into a striking potency of IMM20184/IMM20253 combination against Omicron variant in vivo, decreasing the virus lung load in Omicron infected hamsters ~100 fold to the level comparable to lower limit of detection for the method. We argue that neutralization potency alone does not account for the overall potency in vivo as compared to what was observed for in the published literature. Published data for REGN10933 and REGN10987, the antibodies comprising REGN-CoV2, suggest they are more potent in in vitro neutralization assays (*43*), yet do not appear to lead to higher levels of viral clearance in hamster models of COVID-19 (*44*). We demonstrated that Fc-mediated viral clearance mechanisms were enhanced in the context of the IMM-BCP-01 cocktail as compared to any of the three individual antibodies alone. Thus, we conclude that the enhanced viral clearance observed in vivo may be a direct result of the oligoclonal nature with which the IMM-BCP-01 antibodies bind to the RBD of S protein. The ability to synergistically neutralize via multiple mechanisms, in conjunction with the Fc-dependent combinatorial activation of effector functions, may explain the robust potency detected in the in vivo experiments, and is in agreement with previous reports (*29*–*31*).

Interestingly, no apparent clinical benefit has been derived by increasing the dose of REGN-CoV2 from 2400 mg to 8000 mg (*45*). Similarly, viral clearance of WA1/2020 elicited by VIR-7831 in hamster studies plateaued at approximately 15-fold clearance upon treatment with 5 mg/Kg of antibody; increasing to 15 mg/kg led to no better clearance (*27*). In contrast to those findings, administration of the IMM-BCP-01 cocktail, within the 3 – 9 mg/Kg dose range tested, yielded a dose response of ~300-10,000-fold clearance of the WA1/2020 virus, with the 10,000-fold decrease being at the limit of detection for evaluating absolute clearance levels. Importantly, the dose response was not limited to the WA1/2020 isolate, as a similar dose-response was observed in clearance of Alpha, Beta and Omicron variants. Having the structure and mechanistic data opens up the opportunity for a potential rational design of antibody combinations (*46*, *47*). In summary, we used Immunome’s Discovery platform to identify and characterize three potent patient-derived antibodies IMM20190, IMM20184 and IMM20253 based on the antibody function, epitopes and biochemical properties. When combined into the IMM-BCP-01 cocktail, they synergized to neutralize multiple SARS-CoV-2 variants of concern, potently activate Fc-mediated antiviral effector response, and demonstrate antiviral effects in vivo. We described IMM20253, that recognizes a rare epitope and triggers dissociation of Trimer. Based upon the data we have presented, the IMM-BCP-01 cocktail is effective across the spectrum of variants known to date. Importantly, recent data support the idea that targeting SARS-CoV-2 with several antibodies should reduce viral escape from IMM-BCP-01 (*11*). As the IMM-BCP-01 antibody cocktail may be used in both prophylaxis or therapeutic setting against SARS-CoV-2 variants, clinical trials are warranted.

## MATERIAL AND METHODS

### Study Design

This study describes the identification of patient-derived broadly neutralizing anti-SARS-CoV-2 antibodies using Immunome’s Discovery Platform. The study was aimed to identify a cocktail of anti-SARS-CoV-2 therapeutic antibodies that demonstrate a broad anti-viral neutralization and effector function response and retains therapeutic effect against current and prospective viral variants. Selected antibodies were evaluated in neutralization assays against current VOCs, including Omicron BA.1 and BA.2, in various effector functional assays in vitro and tested in hamsters infected with WA1/2020, Alpha, Beta and Omicron BA.1 variants in vivo. Biochemical mechanistic studies focused on the antibody interactions with viral Spike trimer protein were confirmed by cryo-EM analysis.

### Cells

Reporter virus particles (RVP’s) were purchased from Integral Molecular, ACE2-293T cells (Integral Molecular; Cat #C-HA102) were cultured in DMEM containing 10% FBS, 10mM HEPES, and 0.5 μg/mL Puromycin. Vero E6 cells (BEI resources, NIAID, NIH: VERO C1008 (E6), African green monkey kidney, Working Cell Bank NR-596) were maintained in humidified incubators at 37°C and 5% CO_2_ in DMEM high glucose with GlutaMAX and sodium pyruvate (Gibco^TM^, cat #10569) and 10% certified US-origin heat-inactivated fetal bovine serum (Gibco^TM^, cat #10082). African green monkey Vero-TMPRSS2 (*48*) cells were cultured at 37°C in Dulbecco’s Modified Eagle medium (DMEM) supplemented with 10% fetal bovine serum (FBS), 10 mM HEPES pH 7.3, 1 mM sodium pyruvate, 1× non-essential amino acids, and 100 U/mL of penicillin–streptomycin with 5 μg/mL of blasticidin.

### Virus isolates and reagents

The following reagents were obtained through BEI Resources, NIAID, NIH: VERO C1008 (E6), Kidney (African green monkey), Working Cell Bank, NR-596; SARS-CoV-2, Isolate hCoV-19/USA/CA_CDC_5574/2020, NR-54011 (deposited by Centers for Disease Control and Prevention) and SARS-CoV-2, Isolate hCoV-19/South Africa/KRISP-K005325/2020, NR-54009 (contributed by Alex Sigal and Tulio de Oliveira). The SARS-CoV-2 isolate USA-WA1/2020 starting material was provided by the World Reference Center for Emerging Viruses and Arboviruses (WRCEVA), with Natalie Thornburg (nax3@cdc.gov) as the CDC Principal Investigator.

### Animal studies

All animal studies described in the manuscript were carried out under Institutional Animal Care and Use Committee (IACUC)-approved protocols at the respective institutions (BU and MRIGlobal) and where appropriate were reviewed and approved by Animal Care and Use Review Office of USAMRDC (ACURO).

### Syrian hamster model of SARS-CoV-2 infection

Syrian Golden Hamsters (Envigo) were challenged on Study Day 0 with SARS-CoV-2 via intranasal inoculation using 0.1 mL of either 1.67 × 10^5^ or 1.67 × 10^6^ TCID_50_/mL (WA_CDC-WA1/2020) material (post-exposure treatment experiment), or 1 × 10^4^PFU (WA_CDC-WA1/2020, Alpha (B.1.1.7), Beta (B.1.351), Omicron BA.1) material (pre-exposure treatment experiment). Hamsters were either treated one day before challenge, or 6 ± 1 hour after challenge via an intraperitoneal (i.p.) injection. Animals were euthanized on Study Day 4. The lungs were harvested and homogenized for viral titer determination via TCID_50_ or plaque assay. Viral clearance levels obtained by the various treatments were compared to non-treated controls using a two-way ANOVA with Tukey’s multiple comparisons test. F-tests were performed using an online calculator (https://www.statskingdom.com/220VarF2.html).

### Pseudovirus Production and Neutralization Assay

Neutralization experiments using SARS-CoV-2 luciferase reporter virus particles (RVP’s) (Integral Molecular) were based on the manufacturer’s instructions. In brief, RVP’s were thawed for 2-3 min in a 37°C water bath. The recommended amount of RVP’s was added to the inner wells of a white opaque 96 well plate (Corning; Cat #3917) or 384 well plate (Greiner Bio-One; Cat #781080). Media containing the indicated amount of antibody was added to each well, resulting in a final volume of 100 μL per well (96 well plate) or 25 μL per well (384 well plate). The antibody/RVP mixture was pre-incubated for 1 hour in a 37°C incubator containing 5% CO_2_. ACE2-293T target cells were added to each well (2 × 10^4^ cells in 100 μL for a 96 well plate or 0.9 × 10^4^ cells for a 384 well plate) and incubated for 72 hours. Media was removed from all wells, equal volumes of PBS and Renilla-Glo Luciferase Assay Reagent (Promega; Cat #E2720) were added to each well (60 μL total for a 96 well plate or 30 μL total for a 384 well plate). After 10 min, luminescence was measured on the EnSpire Plate Reader (PerkinElmer). Percent neutralization was calculated with the following equation: [(RLU of Virus + cells) – (RLU of Experimental Sample)] / [(RLU of Virus + cells) – (RLU of cells only)].

### Epitope Mapping of IMM20190, IMM20184 and IMM20253 antibodies

Shotgun Mutagenesis epitope mapping services were provided by Integral Molecular (Philadelphia, PA) as described in (*49*). Briefly, a mutation library of the target protein was created by high-throughput, site-directed mutagenesis. Each residue was individually mutated to alanine, with alanine codons mutated to serine. The mutant library was arrayed in 384-well microplates and transiently transfected into HEK293T cells. Following transfection, cells were incubated with the indicated antibodies at concentrations pre-determined using an independent immunofluorescence titration curve on wild type protein. MAbs were detected using an Alexa Fluor 488-conjugated secondary antibody and mean cellular fluorescence was determined using Intellicyt iQue flow cytometry platform. Mutated residues were identified as being critical to the antibody epitope if they did not support the reactivity of the test antibody but did support the reactivity of the reference MAb. This counter-screen strategy facilitates the exclusion of mutants that are locally misfolded or that have an expression defect.

### Calculation of Synergistic Neutralization by Antibody Combinations

In the context of pseudovirus neutralization, synergy between two or three monoclonal antibodies in combination is defined as neutralization that is greater than neutralization by the most effective monoclonal antibody alone. To test whether combinations of antibodies show synergistic combinatorial effect in neutralizing SARS-CoV-2, we used an approach similar to one described previously (3). Pseudovirus neutralization experiments were set up as described above, except that multiple monoclonal antibodies were tested in combination. Briefly, for combinations of two antibodies, one test article was titrated in the background of each concentration in a serial dilution of the other test article. Single antibody titrations were included as controls. For combinations of three antibodies, one test article was titrated in the background of each concentration in a serial dilution of a 1:1 mixture of the other two test articles. To evaluate antibody synergy in the combinations, the observed combination response matrix of pseudovirus neutralization was used as input for the online SynergyFinder platform (4), where quadruplicate data points were input separately. The highest single agent (HSA) reference model was applied, which quantifies synergy as the excess over the maximum response of a single drug in the combination. Synergy between antibodies in each combination is reported as an overall synergy score (the average of observed synergy across the dose combination matrix) as well as a peak HSA score (the highest synergy score calculated across the dose combination matrix). Synergy scores of less than -10, between -10 and 10, and greater than 10 indicate antagonistic, additive, and synergistic antibody combinations, respectively. While peak HSA scores report on synergy at the most optimal combination concentrations, the overall synergy score is less affected by outlier data points.

### Phagocytosis assay

Assay was performed with antibodies diluted to 100 ug/mL in PBS + 1% BSA. Antibodies were subjected to overnight incubation on tube rotator at 4°C in the presence of bead-biotinylated antigen mixture, followed by 3 washes. THP-1 cells were pelleted, resuspended in serum-free RPM and then added to wells containing bead-antigen-antibody mixture. The bead-antigen-antibody-cells mixture was incubated with cells in CO2 incubator for 18 hours. After that, cells were fixed and immunostained. Flow cytometry was peformed on Attune NXT and the resulted data were analyzed using FlowJo Software.

### Activation of classical complement pathway

ELISA-based method to evaluate the activation of the classical complement pathway was adapted from (*33*, *50*). Endotoxin-free ELISA plates were coated with wither RBD or Trimer soluble proteins diluted in endotoxin-free PBS (HyClone) overnight. Plates were blocked with endotoxin-free 2% gelatin solution (Sigma) and incubated with anti-Spike antibodies of interest for 1 hour at +4°C. Plates were washed 3 times with endotoxin-free GVB buffer with Ca^2+^ and Mg^2+^ (GVB++, Complement Technology) and incubated with normal human serum (Complement Technology) diluted to 1.25% in GVB++ buffer for 1.5 hours at +37°C on an orbital shaker. Reaction was stopped by a wash with ice-cold PBS. Cells with deposited complement components were stained with anti-C4 antisera (Complement Technology) and a secondary anti-goat-HRP antibody (SouthernBiotech). Plates were incubated with HRP substrate and a stop solution according to manufacturer’s instructions (ThermoFisher). Optical density was measured on EnSpire Plate Reader (PerkinElmer)

### Receptor competition assay

ELISA plates were coated with either REF, UK or SA variant of RBD (SinoBio) overnight and washed with PBS (3x). Single antibody or a three-antibody cocktail added at equimolar concentrations were added simultaneously with soluble human ACE2 protein (at a concentration of ~ EC80 of its normal binding to RBD protein) and incubated at +37C on an orbital shaker for 1 hour. Plates were washed (3x) and subsequently probed for ACE2 binding with anti-ACE2 antibody.

### Authentic virus neutralization assay

Antibody combinations starting at 30 μg/ml per antibody were serially diluted in Dulbecco’s Phosphate Buffered Saline (DPBS)(Gibco) using half-log dilutions. Dilutions were prepared in triplicate for each antibody and plated in triplicate. Each dilution was incubated at 37°C and 5% CO_2_ for 1 hour with 10^3^ plaque forming units/ml (PFU/ml) of each SARS-CoV-2 variant [isolate USAWA1/2020, hCoV-19/USA/CA_CDC_5574/2020, BEI #NR-54011 (B.1.1.7) and hCoV-19/South Africa/KRISP-K005325/2020, BEI #NR-54009 (B.1.351) and Omicron BA.1]. Each virus stock was passaged once from starting material in Vero E6 cells prior to use. Controls included Dulbecco’s Modified Eagle Medium (DMEM) (Gibco) containing 2% fetal bovine serum (Gibco) and antibiotic-antimycotic (Gibco) only as a negative control and 1000 PFU/ml SARS-CoV-2 incubated with DPBS. Two hundred microliters of each dilution or control were added to confluent monolayers of NR‐596 Vero E6 cells in duplicate and incubated for 1 hour at 37°C and 5% CO_2_. The plates were gently rocked every 15 min to prevent monolayer drying. The monolayers were then overlaid with a 1:1 solution of 2.5% Avicel® RC‐591 microcrystalline cellulose and carboxymethylcellulose sodium (DuPont Nutrition & Biosciences, Wilmington, DE) and 2X Modified Eagle Medium (Temin’s modification, Gibco) supplemented with 2X antibiotic‐antimycotic (Gibco), 2X GlutaMAX (Gibco) and 10% fetal bovine serum (Gibco). Plates were incubated at 37°C and 5% CO2 for 2 days. The monolayers were fixed with 10% neutral buffered formalin and stained with 0.2% aqueous Gentian Violet (RICCA Chemicals, Arlington, TX) in 10% neutral buffered formalin for 30 min, followed by rinsing and plaque counting. The half maximal inhibitory concentrations (IC50) were calculated using GraphPad Prism 8.

*Focus reduction neutralization assay.* Serial dilutions of antibodies were incubated with 10^2^ focus-forming units (FFU) of WA1/2020 D614G, BA.1, or BA.1.1. for 1 hour at 37°C. Antibody-virus complexes were added to Vero-TMPRSS2 cell monolayers in 96-well plates and incubated at 37°C for 1 hour. Subsequently, cells were overlaid with 1% (w/v) methylcellulose in MEM. Plates were harvested 30 hours (WA1/2020 D614G) or 72 hours (BA.1 and BA.1.1) later by removing overlays and fixed with 4% PFA in PBS for 20 min at room temperature. Plates were washed and sequentially incubated with an oligoclonal pool (SARS2-02, -08, -09, -10, -11, -13, -14, -17, -20, -26, -27, -28, -31, -38, -41, -42, -44, -49,, -57, -62, -64, -65, -67, and -71 (*39*) of anti-S murine antibodies (including cross-reactive mAbs to SARS-CoV) and HRP-conjugated goat anti-mouse IgG (Sigma Cat # A8924) in PBS supplemented with 0.1% saponin and 0.1% bovine serum albumin. SARS-CoV-2-infected cell foci were visualized using TrueBlue peroxidase substrate (KPL) and quantitated on an ImmunoSpot microanalyzer (Cellular Technologies).

### Cryo-EM analysis of Trimer-Fab complexes

Cryo-EM analysis was performed at NovAliX (Strasbourg, France). Fabs were mixed with the SARS-CoV-2 S 6P trimer (6:1 molar ratio Fab per protomer) to a final Fab–S complex concentration of around 0.8 mg ml^−1^ and incubated at room temperature for 1H. Immediately before deposition of 3.5 μl of complex onto a 200 mesh, 1.2/1.3 C-Flat grid (protochips) that had been freshly glow-discharged for 30 s at 3 mA using an ELMO (Cordouan). The sample was incubated on the grid for 15 sec and then blotted with filter paper for 2 s in a temperature and humidity controlled Vitrobot Mark IV (T = 6 °C, humidity 100%, blot force 2) followed by vitrification in 100% liquid ethane. Single-particle cryo-EM data were collected on a Glacios transmission electron microscope (Thermo Fisher) operating at 200 kV. Movies were collected using EPU software for automated data collection. Data were collected at a nominal underfocus of −0.6 to −2.8 μm, at magnifications of 120,000× with a pixel size of 1.2 Å. Micrographs were recorded as movie stacks on a Falcon III direct electron detector (Thermo Fisher); each movie stack was fractionated into 13 frames, for a total exposure of 1.5 s corresponding to an electron dose of 50 e−/Å2. Drift and gain correction and dose weighting were performed using MotionCorr2. A dose-weighted average image of the whole stack was used to determine the contrast transfer function with the software Gctf. The following workflow was processed using RELION 4.0. Ab-initio cryo-EM reconstruction was low-pass filtered to 60 Å and used as an initial reference for 3D classification. The following subclasses depicting high resolution features were selected for refinement with various number of particles. IMM202190: 3 from 8 subclasses, 173,541 particles; IMM20184 2 from 6 subclasses, 62,150 particles; IMM20253: 2 from 6 subclasses for Trimer, 86,974 particles, and 1 from 6 for monomers, 40,489 particles. Atomic models from PDB:7E8C, PDB:6XLU, PDB:6XM5 or PDB:7NOH for the Spike Trimer and PDB:6TCQ for the Fabs were used as starting point. Models were then rigid body fitted to the density in Chimera.

### Cryo-EM analysis of IMM20184/253 Fabs-RBD complex

Cryo-EM analysis was performed at nanoimaging Services (San Diego, USA). Electron microscopy was performed using (1) FEI Titan Krios (Hillsboro, Oregon) transmission electron microscope operated at 300kV and equipped with a Gatan BioQuantum 1967 imaging filter and Gatan K3 Summit direct detector and (2) Thermo Fisher Scientific Glacios Cryo Transmission-electron microscope (Cryo-TEM) operated at 200 kV and equipped with a TFS CETA-D CMOS camera and a Falcon 4 direct electron detector. For Titan Krios, vitreous ice grids were clipped into cartridges, transferred into a cassette and then into the Krios autoloader, all while maintaining the grids at cryogenic temperature (below -170C°). For Glacious Cryo-TEM, vitreous ice grids were clipped into cartridges, transferred into a cassette and then into the Glacios autoloader, all while maintaining the grids at cryogenic temperature (below -170°C). Automated data-collection was carried out using Leginon software (*46*), where high magnification movies were acquired by selecting targets at a lower magnification (*51*). Dose-weighted movie frame alignment was done using MotionCor2 (*52*) or Full-frame or Patch motion correction in cryoSPARC (*53*) to account for stage drift and beam-induced motion. The contrast transfer function is estimated for each micrograph using CTFfind4, gCTF, or Patch CTF in cryoSPARC (*54*, *55*). Individual particles are selected using automated picking protocols and extracted into particle stacks in either Relion (*56*, *57*) or cryoSPARC. The particles may then be submitted to reference-free 2D alignment and classification in either Relion or cryoSPARC. Extracted particles and/or 2D classes showing intact particles are subjected to 2D and/or 3D classification in CryoSPARC 3.1 (*53*). Initial models can be generated *ab initio* from all selected particles, or a suitable model can be imported and used for further 3D classification analysis. The best 3D classes are submitted to homogeneous 3D refinement that includes dynamic masking. Reported resolutions are based on the gold standard FSC = 0.143 criterion (*56*). Maps are visualized using Chimera (*58*). Three datasets were collected. One dataset was collected for sample RBD + IMM20253 Fab + IMM20184 Fab, totaling 3,148 high magnification images. About 1.4M particles were selected from 1,666 manually curated micrographs using cryoSPARC 3.3 live. All subsequent data processing was carried out in cryoSPARC 3.3. These particles were subjected to three rounds of 2D classification, and about 300k good particles were selected. Second dataset with 30° tilt was collected for sample RBD + IMM20253 Fab + IMM20184 Fab, totaling 629 high magnification images. About 100k particles were selected from 244 manually curated micrographs using cryoSPARC 3.3 live. Third dataset with 30° tilt was collected for sample RBD + IMM20253 Fab + IMM20184 Fab, totaling 1,369 high magnification images. Three separate datasets containing > 10^6^ particles, including untilted, untilted chameleon and tilted, were combined for further processing in cryoSPARC 3.3. and ~5.3 × 10^5^ particles from a combined dataset were subjected to ab initio 3D reconstruction. The particles were then subjected to two rounds of heterogeneous refinement using the good and junk classes from ab initio reconstruction as reference volume. The good particles were selected and subjected to homogenous refinement and non-uniform refinement. The final reconstruction included ~300k particles and produced a map at 4 Å nominal resolution (Sup. Figure 1F-J). The generic Fab model (PDB ID:1M71) and RBD model (PDB ID: 7JVB) was rigid-fit in the map, followed by manually built the Fab model (variable regions) based on sequences of IMM20253 and IMM20184 Fabs in Coot. The full model of S-RBD with IMM20253 and IMM20184 Fabs (variable regions) was subjected to five rounds of Phenix real space refinement, followed by visual inspection in COOT to improve the agreement of the model against the map.

### Statistics

Statistical analysis in Fig. 2B is Two-Way ANOVA and in Figs. 2C-H is One-way ANOVA using Dunnet’s multiple comparisons test comparing to untreated group (No Rx) using Prism 9. F-tests were performed using an online calculator (https://www.statskingdom.com/220VarF2.html)

## References

[1] Y. Cao , J. Wang , F. Jian , T. Xiao , W. Song , A. Yisimayi , W. Huang , Q. Li , P. Wang , R. An , J. Wang , Y. Wang , X. Niu , S. Yang , H. Liang , H. Sun , T. Li , Y. Yu , Q. Cui , S. Liu , X. Yang , S. Du , Z. Zhang , X. Hao , F. Shao , R. Jin , X. Wang , J. Xiao , Y. Wang , X. S. Xie , Omicron escapes the majority of existing SARS-CoV-2 neutralizing antibodies. Nature 602, 657–663 (2022). 10.1038/s41586-021-04385-3 35016194 PMC8866119

[2] N. Andrews , J. Stowe , F. Kirsebom , S. Toffa , T. Rickeard , E. Gallagher , C. Gower , M. Kall , N. Groves , A.-M. O’Connell , D. Simons , P. B. Blomquist , A. Zaidi , S. Nash , N. Iwani Binti Abdul Aziz , S. Thelwall , G. Dabrera , R. Myers , G. Amirthalingam , S. Gharbia , J. C. Barrett , R. Elson , S. N. Ladhani , N. Ferguson , M. Zambon , C. N. J. Campbell , K. Brown , S. Hopkins , M. Chand , M. Ramsay , J. Lopez Bernal , Covid-19 Vaccine Effectiveness against the Omicron (B.1.1.529) Variant. N. Engl. J. Med. 386, 1532–1546 (2022). 10.1056/NEJMoa2119451 35249272 PMC8908811

[3] R. Rubin , COVID-19 Vaccines vs Variants-Determining How Much Immunity Is Enough. JAMA 325, 1241–1243 (2021). 10.1001/jama.2021.3370 33729423

[4] D. Planas , D. Veyer , A. Baidaliuk , I. Staropoli , F. Guivel-Benhassine , M. M. Rajah , C. Planchais , F. Porrot , N. Robillard , J. Puech , M. Prot , F. Gallais , P. Gantner , A. Velay , J. Le Guen , N. Kassis-Chikhani , D. Edriss , L. Belec , A. Seve , L. Courtellemont , H. Péré , L. Hocqueloux , S. Fafi-Kremer , T. Prazuck , H. Mouquet , T. Bruel , E. Simon-Lorière , F. A. Rey , O. Schwartz , Reduced sensitivity of SARS-CoV-2 variant Delta to antibody neutralization. Nature 596, 276–280 (2021). 10.1038/s41586-021-03777-9 34237773

[5] J. Lopez Bernal , N. Andrews , C. Gower , E. Gallagher , R. Simmons , S. Thelwall , J. Stowe , E. Tessier , N. Groves , G. Dabrera , R. Myers , C. N. J. Campbell , G. Amirthalingam , M. Edmunds , M. Zambon , K. E. Brown , S. Hopkins , M. Chand , M. Ramsay , Effectiveness of Covid-19 Vaccines against the B.1.617.2 (Delta) Variant. N. Engl. J. Med. 385, 585–594 (2021). 10.1056/NEJMoa2108891 34289274 PMC8314739

[6] N. S. Crowcroft , N. P. Klein , A framework for research on vaccine effectiveness. Vaccine 36, 7286–7293 (2018). 10.1016/j.vaccine.2018.04.016 30431002

[7] M. J. Joyner , R. E. Carter , J. W. Senefeld , S. A. Klassen , J. R. Mills , P. W. Johnson , E. S. Theel , C. C. Wiggins , K. A. Bruno , A. M. Klompas , E. R. Lesser , K. L. Kunze , M. A. Sexton , J. C. Diaz Soto , S. E. Baker , J. R. A. Shepherd , N. van Helmond , N. C. Verdun , P. Marks , C. M. van Buskirk , J. L. Winters , J. R. Stubbs , R. F. Rea , D. O. Hodge , V. Herasevich , E. R. Whelan , A. J. Clayburn , K. F. Larson , J. G. Ripoll , K. J. Andersen , M. R. Buras , M. N. P. Vogt , J. J. Dennis , R. J. Regimbal , P. R. Bauer , J. E. Blair , N. S. Paneth , D. Fairweather , R. S. Wright , A. Casadevall , Convalescent Plasma Antibody Levels and the Risk of Death from Covid-19. N. Engl. J. Med. 384, 1015–1027 (2021). 10.1056/NEJMoa2031893 33523609 PMC7821984

[8] L. M. Katz , (A Little) Clarity on Convalescent Plasma for Covid-19. N. Engl. J. Med. 384, 666–668 (2021). 10.1056/NEJMe2035678 33440086 PMC7821982

[9] M. A. Thompson , J. P. Henderson , P. K. Shah , S. M. Rubinstein , M. J. Joyner , T. K. Choueiri , D. B. Flora , E. A. Griffiths , A. P. Gulati , C. Hwang , V. S. Koshkin , E. B. Papadopoulos , E. V. Robilotti , C. T. Su , E. M. Wulff-Burchfield , Z. Xie , P. P. Yu , S. Mishra , J. W. Senefeld , D. P. Shah , J. L. Warner ; COVID-19 and Cancer Consortium , Association of Convalescent Plasma Therapy With Survival in Patients With Hematologic Cancers and COVID-19. JAMA Oncol. 7, 1167 (2021). 10.1001/jamaoncol.2021.1799 34137799 PMC8377563

[10] K. Garber , Hunt for improved monoclonals against coronavirus gathers pace. Nat. Biotechnol. 39, 9–12 (2021). 10.1038/s41587-020-00791-6 33432224

[11] R. Copin , A. Baum , E. Wloga , K. E. Pascal , S. Giordano , B. O. Fulton , A. Zhou , N. Negron , K. Lanza , N. Chan , A. Coppola , J. Chiu , M. Ni , Y. Wei , G. S. Atwal , A. R. Hernandez , K. Saotome , Y. Zhou , M. C. Franklin , A. T. Hooper , S. McCarthy , S. Hamon , J. D. Hamilton , H. M. Staples , K. Alfson , R. Carrion Jr ., S. Ali , T. Norton , S. Somersan-Karakaya , S. Sivapalasingam , G. A. Herman , D. M. Weinreich , L. Lipsich , N. Stahl , A. J. Murphy , G. D. Yancopoulos , C. A. Kyratsous , The monoclonal antibody combination REGEN-COV protects against SARS-CoV-2 mutational escape in preclinical and human studies. Cell 184, 3949–3961.e11 (2021). 10.1016/j.cell.2021.06.002 34161776 PMC8179113

[12] P. Cavazzoni, FDA Statement: Coronavirus (COVID-19) Update: FDA Limits Use of Certain Monoclonal Antibodies to Treat COVID-19 Due to the Omicron Variant (2022) (available at https://www.fda.gov/news-events/press-announcements/coronavirus-covid-19-update-fda-limits-use-certain-monoclonal-antibodies-treat-covid-19-due-omicron).

[13] CDC, COVID Data Tracker (2022).

[14] G. Vogel , New subvariants are masters of immune evasion. Science 376, 679–680 (2022). 10.1126/science.adc9448 35549399

[15] FDA authorizes revisions to Evusheld dosing | FDA (available at https://www.fda.gov/drugs/drug-safety-and-availability/fda-authorizes-revisions-evusheld-dosing).

[16] Coronavirus (COVID-19) Update: FDA Authorizes New Monoclonal Antibody for Treatment of COVID-19 that Retains Activity Against Omicron Variant | FDA (available at https://www.fda.gov/news-events/press-announcements/coronavirus-covid-19-update-fda-authorizes-new-monoclonal-antibody-treatment-covid-19-retains).

[17] S. Zhou , C. S. Hill , S. Sarkar , L. V. Tse , B. M. D. Woodburn , R. F. Schinazi , T. P. Sheahan , R. S. Baric , M. T. Heise , R. Swanstrom , β-d-N4-hydroxycytidine Inhibits SARS-CoV-2 Through Lethal Mutagenesis But Is Also Mutagenic To Mammalian Cells. J. Infect. Dis. 224, 415–419 (2021). 10.1093/infdis/jiab247 33961695 PMC8136050

[18] D. R. Owen , C. M. N. Allerton , A. S. Anderson , L. Aschenbrenner , M. Avery , S. Berritt , B. Boras , R. D. Cardin , A. Carlo , K. J. Coffman , A. Dantonio , L. Di , H. Eng , R. Ferre , K. S. Gajiwala , S. A. Gibson , S. E. Greasley , B. L. Hurst , E. P. Kadar , A. S. Kalgutkar , J. C. Lee , J. Lee , W. Liu , S. W. Mason , S. Noell , J. J. Novak , R. S. Obach , K. Ogilvie , N. C. Patel , M. Pettersson , D. K. Rai , M. R. Reese , M. F. Sammons , J. G. Sathish , R. S. P. Singh , C. M. Steppan , A. E. Stewart , J. B. Tuttle , L. Updyke , P. R. Verhoest , L. Wei , Q. Yang , Y. Zhu , An Oral SARS-CoV-2 Mpro Inhibitor Clinical Candidate for the Treatment of COVID-19. medRxiv, 2021.07.28.21261232 (2021). 10.1101/2021.07.28.21261232 34726479

[19] FDA, Highlights Of Prescribing Information.

[20] T. Burki , The future of Paxlovid for COVID-19. Lancet Respir. Med. S2213-2600(22)00192-8 (2022). 10.1016/S2213-2600(22)00192-8 35623373 PMC9129253

[21] J. M. DiMuzio , B. C. Heimbach , R. J. Howanski , J. P. Dowling , N. B. Patel , N. Henriquez , C. Nicolescu , M. Nath , A. Polley , J. L. Bingaman , T. Smith , B. C. Harman , M. K. Robinson , M. J. Morin , P. A. Nikitin , Unbiased interrogation of memory B cells from convalescent COVID-19 patients reveals a broad antiviral humoral response targeting SARS-CoV-2 antigens beyond the spike protein. Vaccine X 8, 100098 (2021). 10.1016/j.jvacx.2021.100098 33937741 PMC8064894

[22] Z. Ku , X. Xie , E. Davidson , X. Ye , H. Su , V. D. Menachery , Y. Li , Z. Yuan , X. Zhang , A. E. Muruato , Molecular determinants and mechanism for antibody cocktail preventing SARS-CoV-2 escape. Nat. Commun. 12, 1–13 (2021). 33473140 10.1038/s41467-020-20789-7 PMC7817669

[23] H. Liu , N. C. Wu , M. Yuan , S. Bangaru , J. L. Torres , T. G. Caniels , J. van Schooten , X. Zhu , C. D. Lee , P. J. M. Brouwer , M. J. van Gils , R. W. Sanders , A. B. Ward , I. A. Wilson , Cross-Neutralization of a SARS-CoV-2 Antibody to a Functionally Conserved Site Is Mediated by Avidity. Immunity 53, 1272–1280.e5 (2020). 10.1016/j.immuni.2020.10.023 33242394 PMC7687367

[24] M. Yuan , N. C. Wu , X. Zhu , C. D. Lee , R. T. Y. So , H. Lv , C. K. P. Mok , I. A. Wilson , A highly conserved cryptic epitope in the receptor binding domains of SARS-CoV-2 and SARS-CoV. Science 368, 630–633 (2020). 10.1126/science.abb7269 32245784 PMC7164391

[25] C. G. Rappazzo , L. V. Tse , C. I. Kaku , D. Wrapp , M. Sakharkar , D. Huang , L. M. Deveau , T. J. Yockachonis , A. S. Herbert , M. B. Battles , C. M. O’Brien , M. E. Brown , J. C. Geoghegan , J. Belk , L. Peng , L. Yang , Y. Hou , T. D. Scobey , D. R. Burton , D. Nemazee , J. M. Dye , J. E. Voss , B. M. Gunn , J. S. McLellan , R. S. Baric , L. E. Gralinski , L. M. Walker , Broad and potent activity against SARS-like viruses by an engineered human monoclonal antibody. Science 371, 823–829 (2021). 10.1126/science.abf4830 33495307 PMC7963221

[26] J. A. Jaimes , N. M. André , J. S. Chappie , J. K. Millet , G. R. Whittaker , Phylogenetic Analysis and Structural Modeling of SARS-CoV-2 Spike Protein Reveals an Evolutionary Distinct and Proteolytically Sensitive Activation Loop. J. Mol. Biol. 432, 3309–3325 (2020). 10.1016/j.jmb.2020.04.009 32320687 PMC7166309

[27] A. L. Cathcart, C. Havenar-Daughton, F. A. Lempp, D. Ma, M. Schmid, M. L. Agostini, B. Guarino, J. Di iulio, L. Rosen, H. Tucker, J. Dillen, S. Subramanian, B. Sloan, S. Bianchi, J. Wojcechowskyj, J. Zhou, H. Kaiser, A. Chase, M. Montiel-Ruiz, N. Czudnochowski, E. Cameroni, S. Ledoux, C. Colas, L. Soriaga, A. Telenti, S. Hwang, G. Snell, H. W. Virgin, D. Corti, C. M. Hebner, The dual function monoclonal antibodies VIR-7831 and VIR-7832 demonstrate potent in vitro and in vivo activity against SARS-CoV-2. *bioRxiv*, 2021.03.09.434607 (2021).

[28] A. Ianevski , A. K. Giri , T. Aittokallio , SynergyFinder 2.0: Visual analytics of multi-drug combination synergies. Nucleic Acids Res. 48 (W1), W488–W493 (2020). 10.1093/nar/gkaa216 32246720 PMC7319457

[29] E. S. Winkler , P. Gilchuk , J. Yu , A. L. Bailey , R. E. Chen , Z. Chong , S. J. Zost , H. Jang , Y. Huang , J. D. Allen , J. B. Case , R. E. Sutton , R. H. Carnahan , T. L. Darling , A. C. M. Boon , M. Mack , R. D. Head , T. M. Ross , J. E. Crowe Jr ., M. S. Diamond , Human neutralizing antibodies against SARS-CoV-2 require intact Fc effector functions for optimal therapeutic protection. Cell 184, 1804–1820.e16 (2021). 10.1016/j.cell.2021.02.026 33691139 PMC7879018

[30] A. Schäfer , F. Muecksch , J. C. C. Lorenzi , S. R. Leist , M. Cipolla , S. Bournazos , F. Schmidt , A. Gazumyan , R. S. Baric , D. F. Robbiani , T. Hatziioannou , J. V. Ravetch , P. D. Bieniasz , M. C. Nussenzweig , T. P. Sheahan , Antibody potency, effector function and combinations in protection from SARS-CoV-2 infection in vivo. bioRxiv (2020), doi: . 10.1101/2020.09.15.298067 PMC7673958 33211088

[31] J. Ravetch , R. Yamin , A. Jones , H.-H. Hoffmann , K. Kao , R. Francis , T. Sheahan , R. Baric , C. Rice , S. Bournazos , Fc-engineered antibody therapeutics with improved efficacy against COVID-19. (2021), doi: . 10.21203/rs.3.rs-555612/v1 PMC9038156 34547765

[32] M. E. Ackerman , B. Moldt , R. T. Wyatt , A. S. Dugast , E. McAndrew , S. Tsoukas , S. Jost , C. T. Berger , G. Sciaranghella , Q. Liu , D. J. Irvine , D. R. Burton , G. Alter , A robust, high-throughput assay to determine the phagocytic activity of clinical antibody samples. J. Immunol. Methods 366, 8–19 (2011). 10.1016/j.jim.2010.12.016 21192942 PMC3050993

[33] P. A. Nikitin , E. L. Rose , T. S. Byun , G. C. Parry , S. Panicker , C1s Inhibition by BIVV009 (Sutimlimab) Prevents Complement-Enhanced Activation of Autoimmune Human B Cells In Vitro. J. Immunol. 202, 1200–1209 (2019). 10.4049/jimmunol.1800998 30635392 PMC6360260

[34] G. Wang , R. N. de Jong , E. T. van den Bremer , F. J. Beurskens , A. F. Labrijn , D. Ugurlar , P. Gros , J. Schuurman , P. W. Parren , A. J. Heck , Molecular Basis of Assembly and Activation of Complement Component C1 in Complex with Immunoglobulin G1 and Antigen. Mol. Cell 63, 135–145 (2016). 10.1016/j.molcel.2016.05.016 27320199

[35] C. A. Diebolder , F. J. Beurskens , R. N. de Jong , R. I. Koning , K. Strumane , M. A. Lindorfer , M. Voorhorst , D. Ugurlar , S. Rosati , A. J. R. Heck , J. G. J. van de Winkel , I. A. Wilson , A. J. Koster , R. P. Taylor , E. O. Saphire , D. R. Burton , J. Schuurman , P. Gros , P. W. H. I. Parren , Complement is activated by IgG hexamers assembled at the cell surface. Science 343, 1260–1263 (2014). 10.1126/science.1248943 24626930 PMC4250092

[36] D. Sun , Z. Sang , Y. J. Kim , Y. Xiang , T. Cohen , A. K. Belford , A. Huet , J. F. Conway , J. Sun , D. J. Taylor , D. Schneidman-Duhovny , C. Zhang , W. Huang , Y. Shi , Potent neutralizing nanobodies resist convergent circulating variants of SARS-CoV-2 by targeting diverse and conserved epitopes. Nat. Commun. 12, 4676 (2021). 10.1038/s41467-021-24963-3 34344900 PMC8333356

[37] A. J. Greaney , A. N. Loes , K. H. D. Crawford , T. N. Starr , K. D. Malone , H. Y. Chu , J. D. Bloom , Comprehensive mapping of mutations in the SARS-CoV-2 receptor-binding domain that affect recognition by polyclonal human plasma antibodies. Cell Host Microbe 29, 463–476.e6 (2021). 10.1016/j.chom.2021.02.003 33592168 PMC7869748

[38] J. Zahradník , S. Marciano , M. Shemesh , E. Zoler , J. Chiaravalli , B. Meyer , O. Dym , N. Elad , G. Schreiber , SARS-CoV-2 RBD in vitro evolution follows contagious mutation spread, yet generates an able infection inhibitor. bioRxiv (2021), doi:. 10.1101/2021.01.06.425392

[39] L. A. VanBlargan, L. J. Adams, Z. Liu, R. E. Chen, P. Gilchuk, S. Raju, B. K. Smith, H. Zhao, J. Brett Case, E. S. Winkler, B. M. Whitener, L. Droit, I. D. Aziati, P.-Y. Shi, A. Creanga, A. Pegu, S. A. Handley, D. Wang, A. C. M. Boon, J. E. Crowe Jr., S. P. J. Whelan, D. H. Fremont, M. S. Diamond, A potently neutralizing anti-SARS-CoV-2 antibody inhibits variants of concern by binding a highly conserved epitope. *bioRxiv* (2021), doi: . 10.1101/2021.04.26.441501 PMC8373659 34481543

[40] D. Pinto , Y. J. Park , M. Beltramello , A. C. Walls , M. A. Tortorici , S. Bianchi , S. Jaconi , K. Culap , F. Zatta , A. De Marco , A. Peter , B. Guarino , R. Spreafico , E. Cameroni , J. B. Case , R. E. Chen , C. Havenar-Daughton , G. Snell , A. Telenti , H. W. Virgin , A. Lanzavecchia , M. S. Diamond , K. Fink , D. Veesler , D. Corti , Cross-neutralization of SARS-CoV-2 by a human monoclonal SARS-CoV antibody. Nature 583, 290–295 (2020). 10.1038/s41586-020-2349-y 32422645

[41] A. C. Walls , Y. J. Park , M. A. Tortorici , A. Wall , A. T. McGuire , D. Veesler , Structure, Function, and Antigenicity of the SARS-CoV-2 Spike Glycoprotein. Cell 181, 281–292.e6 (2020). 10.1016/j.cell.2020.02.058 32155444 PMC7102599

[42] C. O. Barnes , C. A. Jette , M. E. Abernathy , K. A. Dam , S. R. Esswein , H. B. Gristick , A. G. Malyutin , N. G. Sharaf , K. E. Huey-Tubman , Y. E. Lee , D. F. Robbiani , M. C. Nussenzweig , A. P. West Jr ., P. J. Bjorkman , SARS-CoV-2 neutralizing antibody structures inform therapeutic strategies. Nature 588, 682–687 (2020). 10.1038/s41586-020-2852-1 33045718 PMC8092461

[43] J. Hansen , A. Baum , K. E. Pascal , V. Russo , S. Giordano , E. Wloga , B. O. Fulton , Y. Yan , K. Koon , K. Patel , K. M. Chung , A. Hermann , E. Ullman , J. Cruz , A. Rafique , T. Huang , J. Fairhurst , C. Libertiny , M. Malbec , W. Y. Lee , R. Welsh , G. Farr , S. Pennington , D. Deshpande , J. Cheng , A. Watty , P. Bouffard , R. Babb , N. Levenkova , C. Chen , B. Zhang , A. Romero Hernandez , K. Saotome , Y. Zhou , M. Franklin , S. Sivapalasingam , D. C. Lye , S. Weston , J. Logue , R. Haupt , M. Frieman , G. Chen , W. Olson , A. J. Murphy , N. Stahl , G. D. Yancopoulos , C. A. Kyratsous , Studies in humanized mice and convalescent humans yield a SARS-CoV-2 antibody cocktail. Science 369, 1010–1014 (2020). 10.1126/science.abd0827 32540901 PMC7299284

[44] A. Baum , D. Ajithdoss , R. Copin , A. Zhou , K. Lanza , N. Negron , M. Ni , Y. Wei , K. Mohammadi , B. Musser , G. S. Atwal , A. Oyejide , Y. Goez-Gazi , J. Dutton , E. Clemmons , H. M. Staples , C. Bartley , B. Klaffke , K. Alfson , M. Gazi , O. Gonzalez , E. Dick Jr ., R. Carrion Jr ., L. Pessaint , M. Porto , A. Cook , R. Brown , V. Ali , J. Greenhouse , T. Taylor , H. Andersen , M. G. Lewis , N. Stahl , A. J. Murphy , G. D. Yancopoulos , C. A. Kyratsous , REGN-COV2 antibodies prevent and treat SARS-CoV-2 infection in rhesus macaques and hamsters. Science 370, 1110–1115 (2020). 10.1126/science.abe2402 33037066 PMC7857396

[45] D. M. Weinreich , S. Sivapalasingam , T. Norton , S. Ali , H. Gao , R. Bhore , B. J. Musser , Y. Soo , D. Rofail , J. Im , C. Perry , C. Pan , R. Hosain , A. Mahmood , J. D. Davis , K. C. Turner , A. T. Hooper , J. D. Hamilton , A. Baum , C. A. Kyratsous , Y. Kim , A. Cook , W. Kampman , A. Kohli , Y. Sachdeva , X. Graber , B. Kowal , T. DiCioccio , N. Stahl , L. Lipsich , N. Braunstein , G. Herman , G. D. Yancopoulos ; Trial Investigators , REGN-COV2, a Neutralizing Antibody Cocktail, in Outpatients with Covid-19. N. Engl. J. Med. 384, 238–251 (2021). 10.1056/NEJMoa2035002 33332778 PMC7781102

[46] Y. Sun , L. Wang , R. Feng , N. Wang , Y. Wang , D. Zhu , X. Xing , P. Yang , Y. Zhang , W. Li , X. Wang , Structure-based development of three- and four-antibody cocktails against SARS-CoV-2 via multiple mechanisms. Cell Res. 31, 597–600 (2021). 10.1038/s41422-021-00497-7 33782529 PMC8005859

[47] H. Yao , Y. Sun , Y.-Q. Deng , N. Wang , Y. Tan , N.-N. Zhang , X.-F. Li , C. Kong , Y.-P. Xu , Q. Chen , T.-S. Cao , H. Zhao , X. Yan , L. Cao , Z. Lv , D. Zhu , R. Feng , N. Wu , W. Zhang , Y. Hu , K. Chen , R.-R. Zhang , Q. Lv , S. Sun , Y. Zhou , R. Yan , G. Yang , X. Sun , C. Liu , X. Lu , L. Cheng , H. Qiu , X.-Y. Huang , T. Weng , D. Shi , W. Jiang , J. Shao , L. Wang , J. Zhang , T. Jiang , G. Lang , C.-F. Qin , L. Li , X. Wang , Rational development of a human antibody cocktail that deploys multiple functions to confer Pan-SARS-CoVs protection. Cell Res. 31, 25–36 (2021). 10.1038/s41422-020-00444-y 33262452 PMC7705443

[48] R. Zang , M. F. Gomez Castro , B. T. McCune , Q. Zeng , P. W. Rothlauf , N. M. Sonnek , Z. Liu , K. F. Brulois , X. Wang , H. B. Greenberg , M. S. Diamond , M. A. Ciorba , S. P. J. Whelan , S. Ding , TMPRSS2 and TMPRSS4 promote SARS-CoV-2 infection of human small intestinal enterocytes. Sci. Immunol. 5, eabc3582 (2020). 32404436 10.1126/sciimmunol.abc3582 PMC7285829

[49] E. Davidson , B. J. Doranz , A high-throughput shotgun mutagenesis approach to mapping B-cell antibody epitopes. Immunology 143, 13–20 (2014). 10.1111/imm.12323 24854488 PMC4137951

[50] J. Shi , E. L. Rose , A. Singh , S. Hussain , N. E. Stagliano , G. C. Parry , S. Panicker , TNT003, an inhibitor of the serine protease C1s, prevents complement activation induced by cold agglutinins. Blood 123, 4015–4022 (2014). 10.1182/blood-2014-02-556027 24695853

[51] C. Suloway , J. Pulokas , D. Fellmann , A. Cheng , F. Guerra , J. Quispe , S. Stagg , C. S. Potter , B. Carragher , Automated molecular microscopy: The new Leginon system. J. Struct. Biol. 151, 41–60 (2005). 10.1016/j.jsb.2005.03.010 15890530

[52] S. Q. Zheng , E. Palovcak , J. P. Armache , K. A. Verba , Y. Cheng , D. A. Agard , MotionCor2: Anisotropic correction of beam-induced motion for improved cryo-electron microscopy. Nat. Methods 14, 331–332 (2017). 10.1038/nmeth.4193 28250466 PMC5494038

[53] A. Punjani , J. L. Rubinstein , D. J. Fleet , M. A. Brubaker , cryoSPARC: Algorithms for rapid unsupervised cryo-EM structure determination. Nat. Methods 14, 290–296 (2017). 10.1038/nmeth.4169 28165473

[54] A. Rohou , N. Grigorieff , CTFFIND4: Fast and accurate defocus estimation from electron micrographs. J. Struct. Biol. 192, 216–221 (2015). 10.1016/j.jsb.2015.08.008 26278980 PMC6760662

[55] K. Zhang , Gctf: Real-time CTF determination and correction. J. Struct. Biol. 193, 1–12 (2016). 10.1016/j.jsb.2015.11.003 26592709 PMC4711343

[56] S. H. W. Scheres, in *Methods in Enzymology*, (2016), vol. 579. 10.1016/bs.mie.2016.04.012 27572726

[57] D. Kimanius , B. O. Forsberg , S. H. W. Scheres , E. Lindahl , Accelerated cryo-EM structure determination with parallelisation using GPUs in RELION-2. eLife 5, e18722 (2016). 10.7554/eLife.18722 27845625 PMC5310839

[58] T. D. Goddard , C. C. Huang , T. E. Ferrin , Visualizing density maps with UCSF Chimera. J. Struct. Biol. 157, 281–287 (2007). 10.1016/j.jsb.2006.06.010 16963278

