# Supplementary material for: IMM-BCP-01, a patient-derived anti-SARS-CoV-2 antibody cocktail, is active across variants of concern including Omicron BA.1 and BA.2
Source: Sci Immunol. 2022 Jun 30:eabl9943. doi: 10.1126/sciimmunol.abl9943 (PMC9273042; doi:10.1126/sciimmunol.abl9943)
Supplement: Supplementary file 1 — Figs. S1 to S5 Table S1 [file sciimmunol.abl9943_sm.pdf]

Science Immunology
AAAS

Supplementary Materials for

IMM-BCP-01, a patient-derived anti-SARS-CoV-2 antibody cocktail, is active across variants of concern including Omicron BA.1 and BA.2

Pavel A. Nikitin *et al.*

Corresponding author: Pavel A. Nikitin, pnikitin@immunome.com

DOI: 10.1126/sciimmunol.abl9943

The PDF file includes:

Figs. S1 to S5
Table S1

Other Supplementary Material for this manuscript includes the following:

Table S2

Supplementary Materials information to IMM-BCP-01, a Patient-Derived Anti-SARS-CoV-2 Antibody Cocktail, Is Active Across Variants of Concern Including Omicron BA.1 and BA.2

Authors: Pavel A. Nikitin[1*], Jillian M. DiMuzio[1], John P. Dowling[1], Nirja B. Patel[1], Jamie L. Bingaman-Steele[1], Baron C. Heimbach[1], Noeleya Henriquez[1], Chris Nicolescu[1], Antonio Polley[1], Eden L. Sikorski[1], Raymond J. Howanski[1], Mitchell Nath[1], Halley Shukla[1], Suzanne M. Scheaffer[3], James P. Finn[1], Li-Fang Liang[1], Todd Smith[1], Nadia Storm[2], Lindsay G. A. McKay[2], Rebecca I. Johnson[2], Lauren E. Malsick[2], Anna N. Honko[2], Anthony Griffiths[2], Michael S. Diamond[3], Purnanand Sarma[1], Dennis H. Geising[1], Michael J. Morin[1], and Matthew K. Robinson[1]

Affiliations:

[1]Immunome, Inc., Exton, PA, U.S.A.

[2]Department of Microbiology, Boston University School of Medicine and National Emerging Infectious Diseases Laboratories, Boston, MA, USA

[3]Departments of Medicine, Molecular Microbiology, Pathology & Immunology, Washington University School of Medicine, St. Louis, MO 63110, USA

***Corresponding author**

Email: pnikitin@immunome.com

LIST OF SUPPLEMENTARY MATERIALS

Supplementary Table 1. Breadth of binding of IMM20190/184/253 antibodies to RBD proteins bearing mutations found in CDC VOCs.

Supplementary Table 2. Raw data.

Supplementary Figure 1. Binding patterns of IMM20184, IMM20190 and IMM20253 Fabs to Spike protein.

Supplementary Figure 2. Antibody exposure and pharmacokinetics in dosed hamsters.

Supplementary Figure 3. Three selected antibodies have a synergistic neutralizing effect.

Supplementary Figure 4. Focus reduction neutralization assay (FRNT) of SARS-CoV-2 variants in the presence of IMM20184, IMM20253 and IMM20253/184 combination.

Supplementary Figure 5. Binding of IMM antibodies to soluble RBD proteins from SARS-CoV-1 and SARS-CoV-2 variants in a steady-state hTRF assay.

Supplementary Tables

Supplementary Table 1. Breadth of binding of IMM20190/184/253 antibodies to RBD proteins bearing mutations found in CDC VOCs. EC50 (pM) relative to respective reference proteins measured using hTRF assay.

Sort	His-tagged protein	Binding Region	Region of Origin	Variant	Lineage	IMM20190	IMM20253	IMM20184
1	A352S	RBD				25.4	33.0	27.7
2	A475V	RBD				70.2	34.6	30.3
3	E406Q	RBD				67.2	44.1	29.2
4	E484K	RBD	South Africa; Brazil	beta; gamm	B.1.351; P.1	39.2	32.7	23.2
5	E484Q	RBD	India		B.1.617	59.9	22.7	21.0
6	F486S	RBD		lambda	C.37	41.2	26.3	23.0
7	F490S	RBD	Peru	lambda	C.37	14.7	73.4	60.8
8	G339D, S371L, S373P, S375F, K417N, N440K, G446S, S477N, T478K, E484A, Q493R, G496S, Q498R, N501Y, Y505H	RBD	South Africa	omicron	B.1.1.529	not detected	48.5	not detected
9	K417N	RBD	South Africa	beta	B.1.351	>500	67.4	44.3
10	K417N, E484K, N501Y	RBD	South Africa	beta	B.1.351	not detected	25.0	20.7
11	K417T, E484K, N501Y	RBD	Brazil	gamma	P.1	>500	22.6	17.9
12	K444R	RBD				33.9	26.7	20.9
13	L452R	RBD	USA; India	epsilon; delt	B.1.427; B.1.617	40.9	34.3	41.2
14	L452R, T478K	RBD	India	delta	B.1.617.2	52.8	61.7	111
15	N439K	RBD	Scotland; Europe			76.6	56.5	52.1
16	N440K	RBD				45.6	39.7	32.1
17	N501Y	RBD	UK; South Africa; Brazil	alpha; beta;	B.1.1.7; B.1.351; P.1	311	59.0	42.2
**18**	**Spike RBD (319-591)**	**RBD**	**Wuhan / Washington reference**	**reference**		**69.9**	**53.4**	**46.2**
19	T478I	RBD				16.4	45.5	68.5
20	Y453F	RBD	Denmark	mink		71.6	65.6	50.1
21	A222S, D614G	S1	Europe; Spain			51.0	45.1	29.9
22	D614G	S1	Multiple			69.9	62.9	44.1
23	K417N, E484K, N501Y, D614G	S1	South Africa	beta	B.1.351	391	34.7	25.6
**24**	**Spike S1 (WT)**	**S1**	**Wuhan / Washington reference**	**reference**		**58.8**	**50.0**	**34.4**
25	SARS-CoV-1	S1	CoV-1 reference	wild type		not detected	137.0	>500
26	T19R, G142D, E156G, Δ157-158, L452R, T478K, D614G, P681R	S1	India	delta	B.1.617.2	22.2	40.9	30.6
27	E154K, L452R, E484Q, D614G, P681R	S1	India		B.1.617	38.2	49.2	22.7
28	ΔHV69/70, 501Y, D614G	S1	UK	alpha	B.1.1.7			
29	ΔHV69/70, Y453F, D614G	S1	Denmark	mink		48.1	67.1	34.8
30	ΔHV69/70, ΔY144, N501Y, A570D, D614G, P681H	S1	UK	alpha	B.1.1.7	>500	63.1	49.1

Supplementary Figures

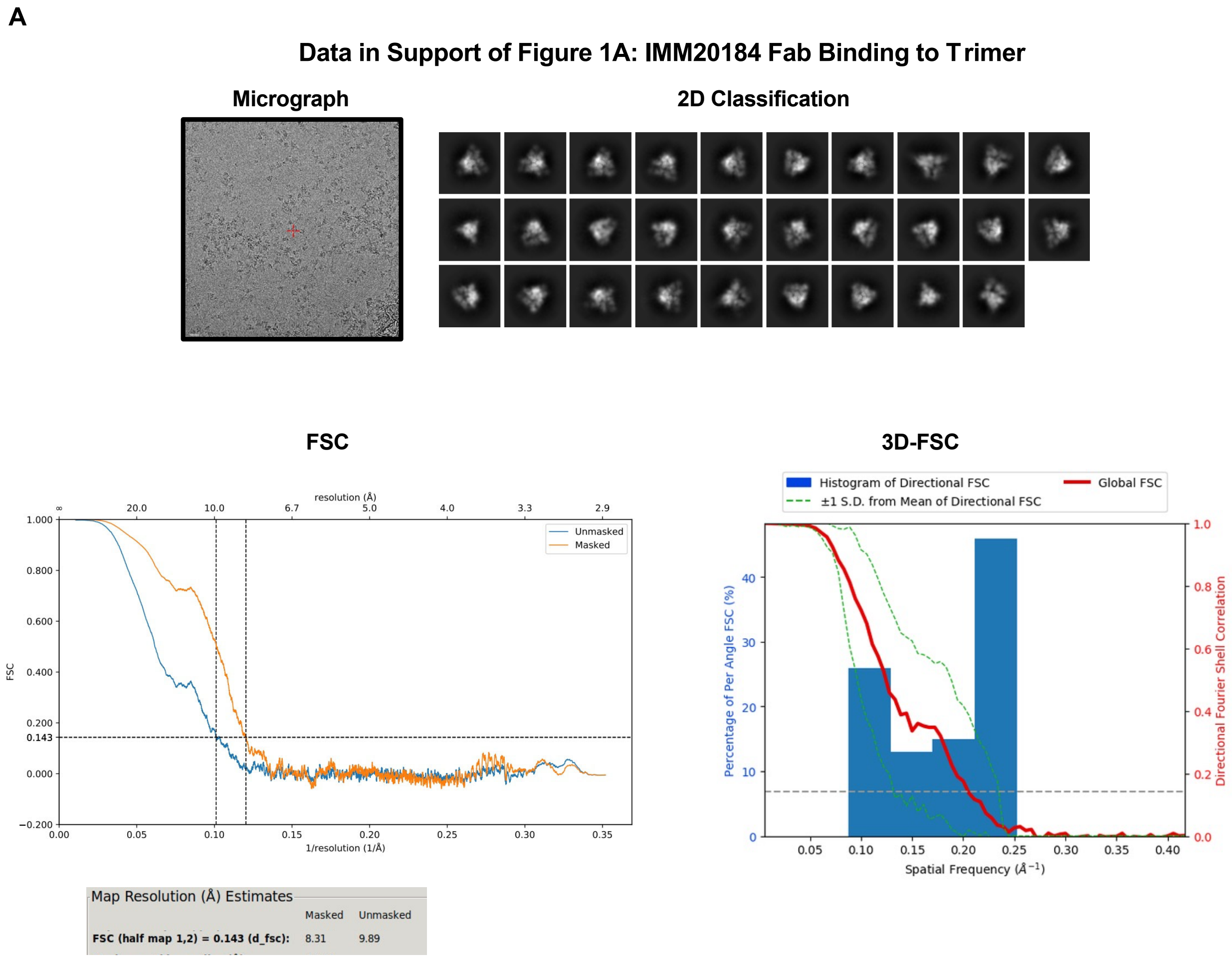

B

Data in Support of Figure 1A: IMM20190 Fab Binding to Trimer

Micrograph

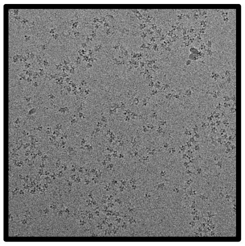

2D Classification

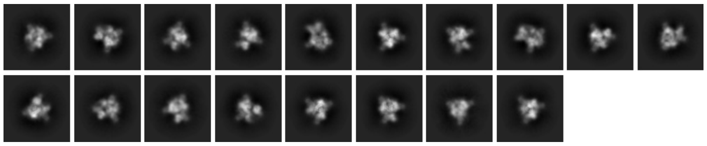

FSC

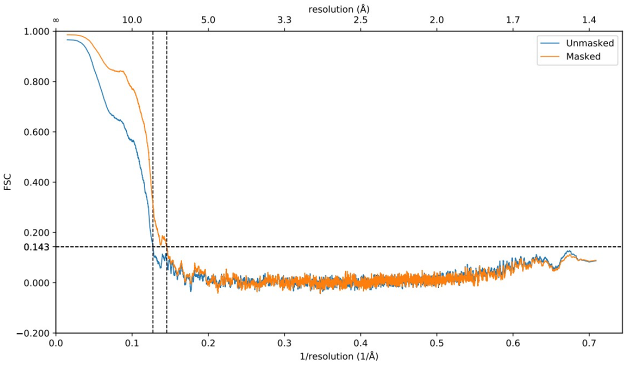

3D-FSC

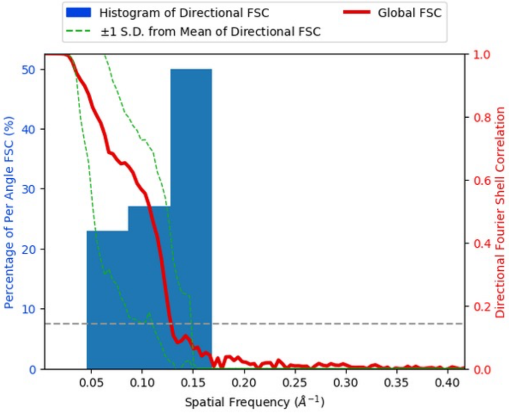

Map Resolution (Å) Estimates

	Masked	Unmasked
**FSC (half map 1,2) = 0.143 (d_fsc):**	6.87	7.85

C Data in Support of Figure 1A: IMM20253 Fab Binding to Trimer

Micrograph

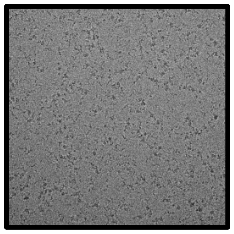

2D Classification

Family #1

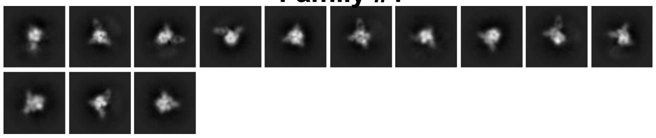

FSC

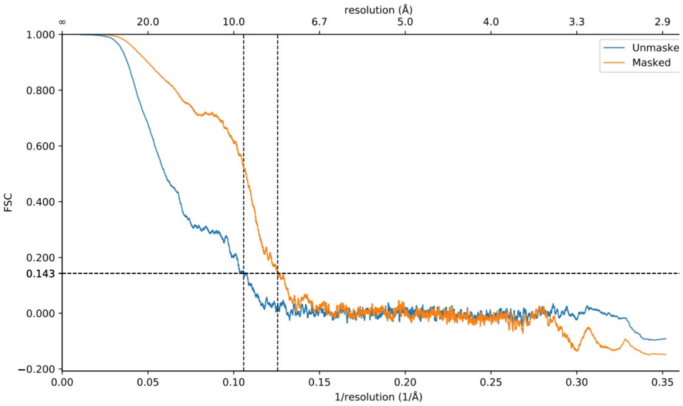

3D-FSC

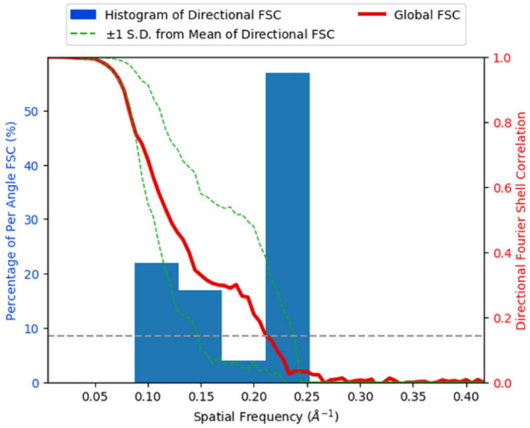

Map Resolution (Å) Estimates	Masked	Unmasked
**FSC (half map 1,2) = 0.143 (d_fsc):**	7.96	9.45

Family #2

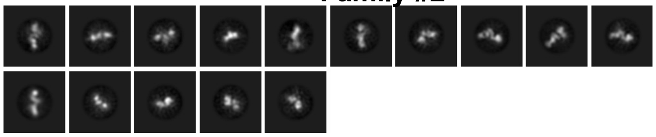

FSC

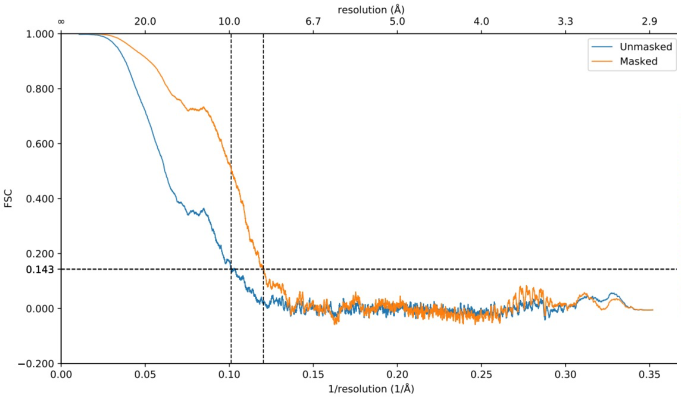

3D-FSC

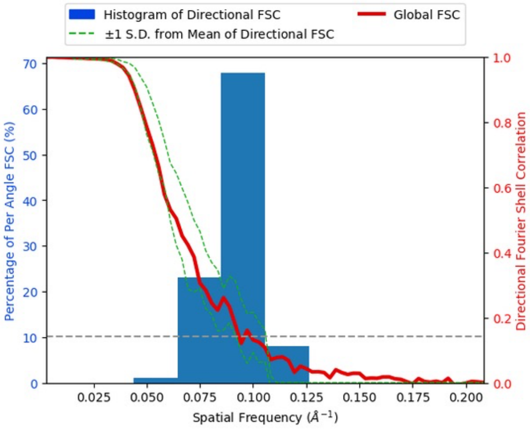

Map Resolution (Å) Estimates	Masked	Unmasked
**FSC (half map 1,2) = 0.143 (d_fsc):**	8.01	10.79

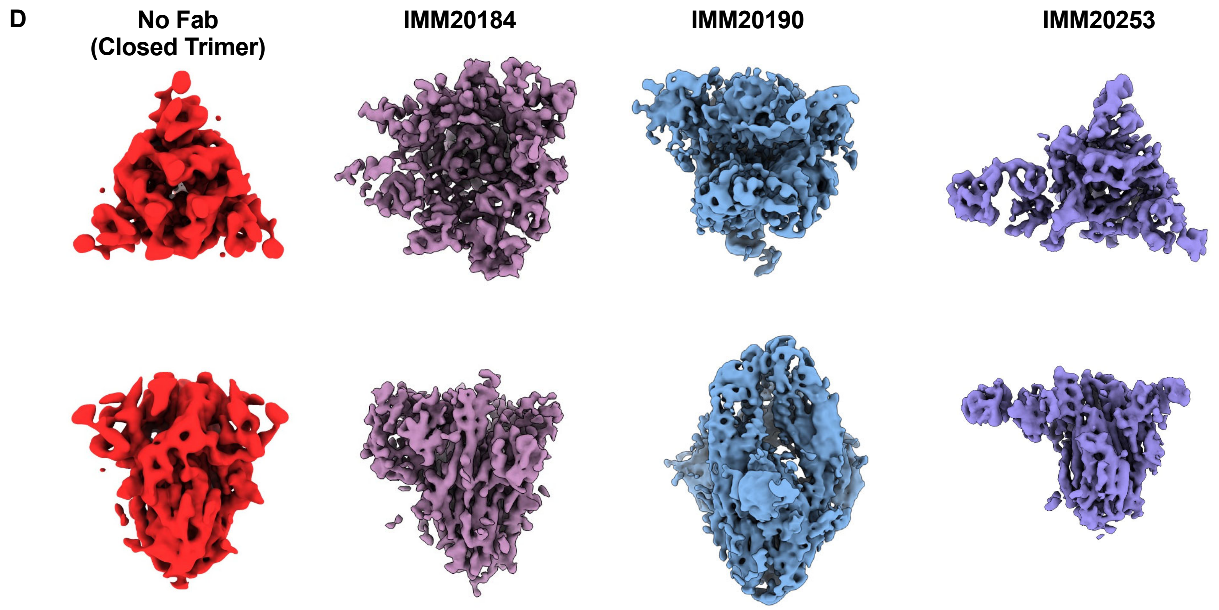
D
No Fab
(Closed Trimer)
IMM20184
IMM20190
IMM20253

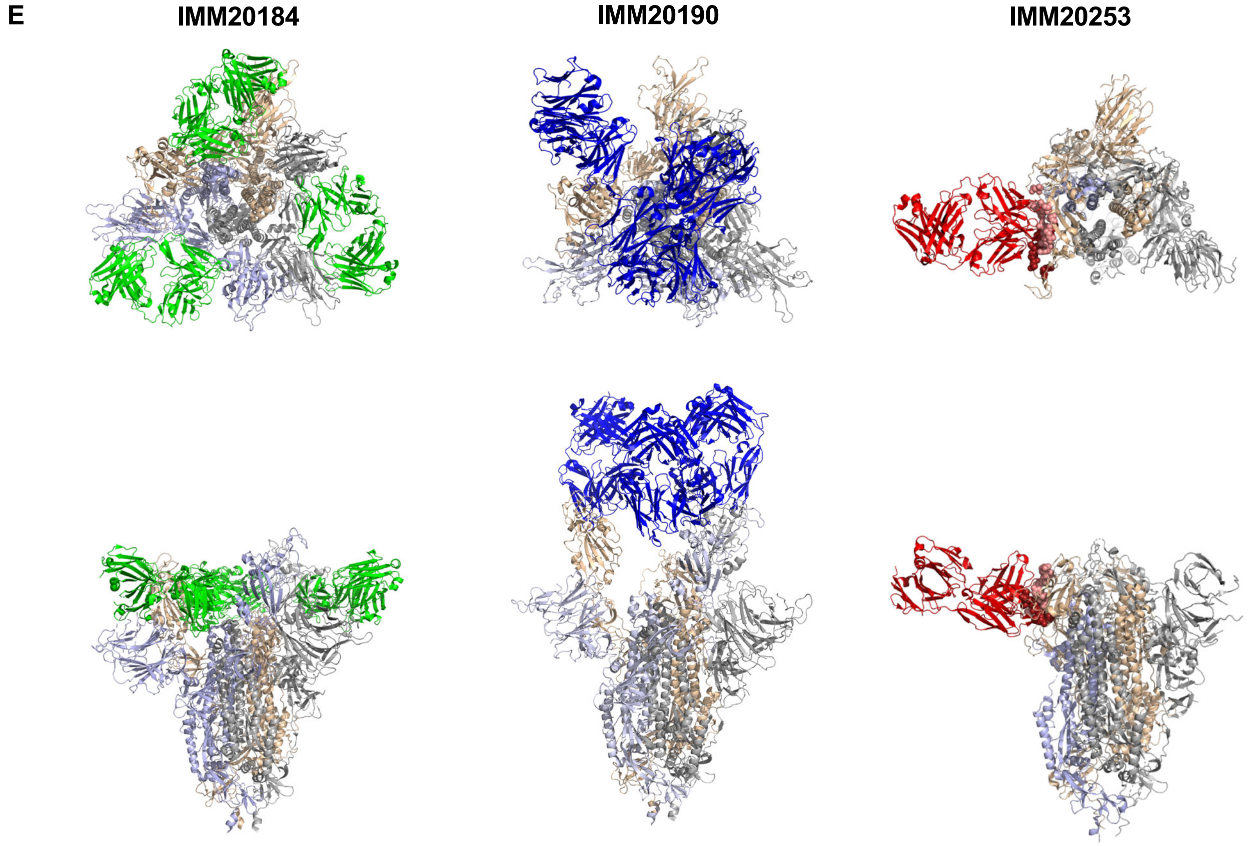
E
IMM20184
IMM20190
IMM20253

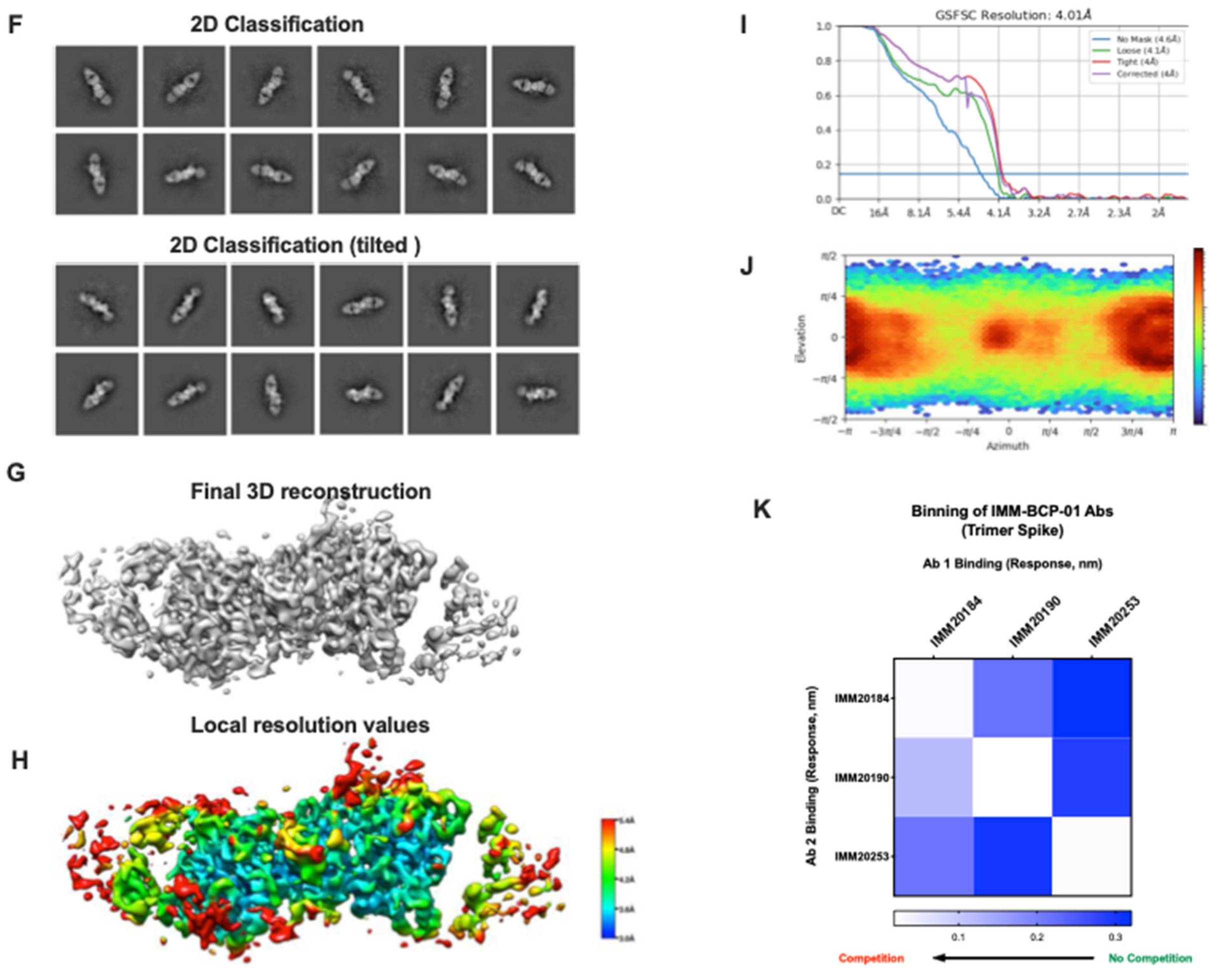

Supplementary Figure 1. Binding pattern of IMM20184, IMM20190 and IMM20253 Fabs to Spike protein. (A) Cryo-EM micrographs, 2D classification and Fourier shell correlation (FSC) and 3D-FSC graphs of Trimer-IMM20184 Fab complex. (B) Cryo-EM micrographs, 2D classification and Fourier shell correlation (FSC) and 3D-FSC graphs of Trimer-IMM20190 Fab complex. (C) Cryo-EM micrographs, 2D classification and Fourier shell correlation (FSC) and 3D-FSC graphs of Trimer-IMM20253 Fab complex.IMM20253 Fab binding to Trimer generates two families (shown as Family#1 and 2). (D). Comparison of 3D reconstruction data (density only) for a closed Trimer conformation, IMM20184 Fab-Trimer, IMM20190 Fab-Trimer and IMM20253 Fab-Trimer complexes in support of data shown in Figure 1A. (E) Models PDB:7E8C,

PDB:6XLU, PDB:6XM5 or PDB:7NOH for Trimer and PDB:6TCQ for the Fabs demonstrate binding patterns and attack angles for IMM20184/190/253 antibodies. (F) CryoEM micrographs and 2D classification of a IMM20184 Fab – RBD – IMM20253 Fab complex for untilted (Top) and tilted (Bottom) datasets. Simultaneous binding of both Fabs is clearly visible. (G). Final 3D reconstruction of data shown in Supp. Figure 1F. Figure is generated in Chimera. (H) Final 4.0 Å map of S-RBD complexed with IMM20253 Fab and IMM20184 colored by the local resolution values as calculated by cryoSPARC 3.3. Figure generated with Chimera. (I) Fourier shell correlation (FSC) curves of the final 3D refinement of data from panel G and H in cryoSPARC 3.3 for different types of masks. (J). Viewing directional distribution for the final refinement run for the complex shown in panels G and G, generated by cryoSPARC 3.3. The viewing direction distribution histogram shows the number of images with a particular viewing direction at each (elevation, azimuth angle). (K). Antibody binning on Octet Qke. IMM20184, IMM20190 and IMM20253 do not compete for soluble Trimer and RBD protein binding. Heat map values represent binding of the first antibody to Trimer (top), followed by binding of the second antibody (left), measured as Response parameter in nm.

A

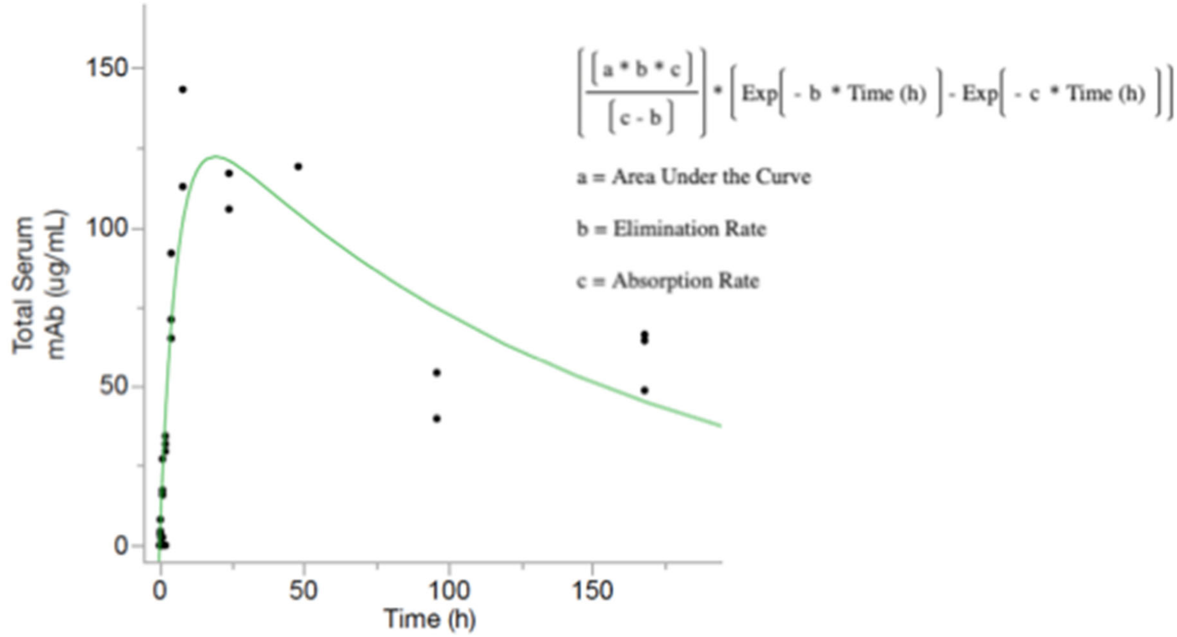

B

	Total mAb Dose (mg/Kg)	C_{max} (µg/mL)	C_96hr (µg/mL)	Absorption Rate (hr^{-1})	Elimination Rate (hr^{-1})	Half-life (hr)	AUC_0-96hr (µg × hr/mL)	AUCinf (µg × hr/mL)
Observed	9	120.5	74.4	0.174	0.007	99.6	9259	20195
Estimated	6	80.3	49.6	0.174	0.007	99.6	6173	13463
Estimated	3	40.2	24.8	0.174	0.007	99.6	3086	6732

C

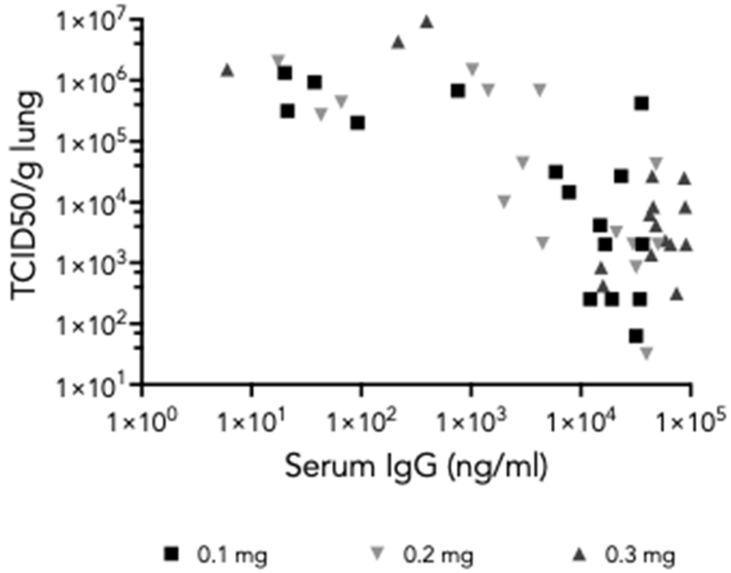

Supplementary Figure 2. Antibody exposure and pharmacokinetics in dosed hamsters. (A) Pharmacokinetics of the 3-Ab cocktail in hamsters. The 3-Ab cocktail (0.3 mg each) was administered i.p. into Syrian Golden hamsters and terminal bleeds (n = 4 per time point) were taken at 0.25, 0.5. 1, 2, 4, 8, 24, 48, 96, and 168 hours post administration. Total human IgG levels were determined by anti-human ELISA. Pharmacokinetics in animals exhibiting < 1000 ng/mL IgG in serum at timepoints >30 minutes post-injection. Green line is the calculated curve using the formula shown on the right. (B) PK parameters of data from panel A. (C) Viral titer in lungs of infected hamsters depends upon Ab exposure. Syrian golden hamsters challenged with $3.3x10^5$ TCID50 viral inoculation of a non-adapted WA_CDC-WA1/2020 SARS-CoV-2 isolate were treated with 3-Ab cocktail (IMM20184/IMM20190/IMM20253), at various dose levels, six hours post inoculation with virus. Lungs were harvested at day 4 post-treatment and viral titers were determined by TCID50 assay. Terminal levels of IgG in blood were quantified by anti-human ELISA.

A

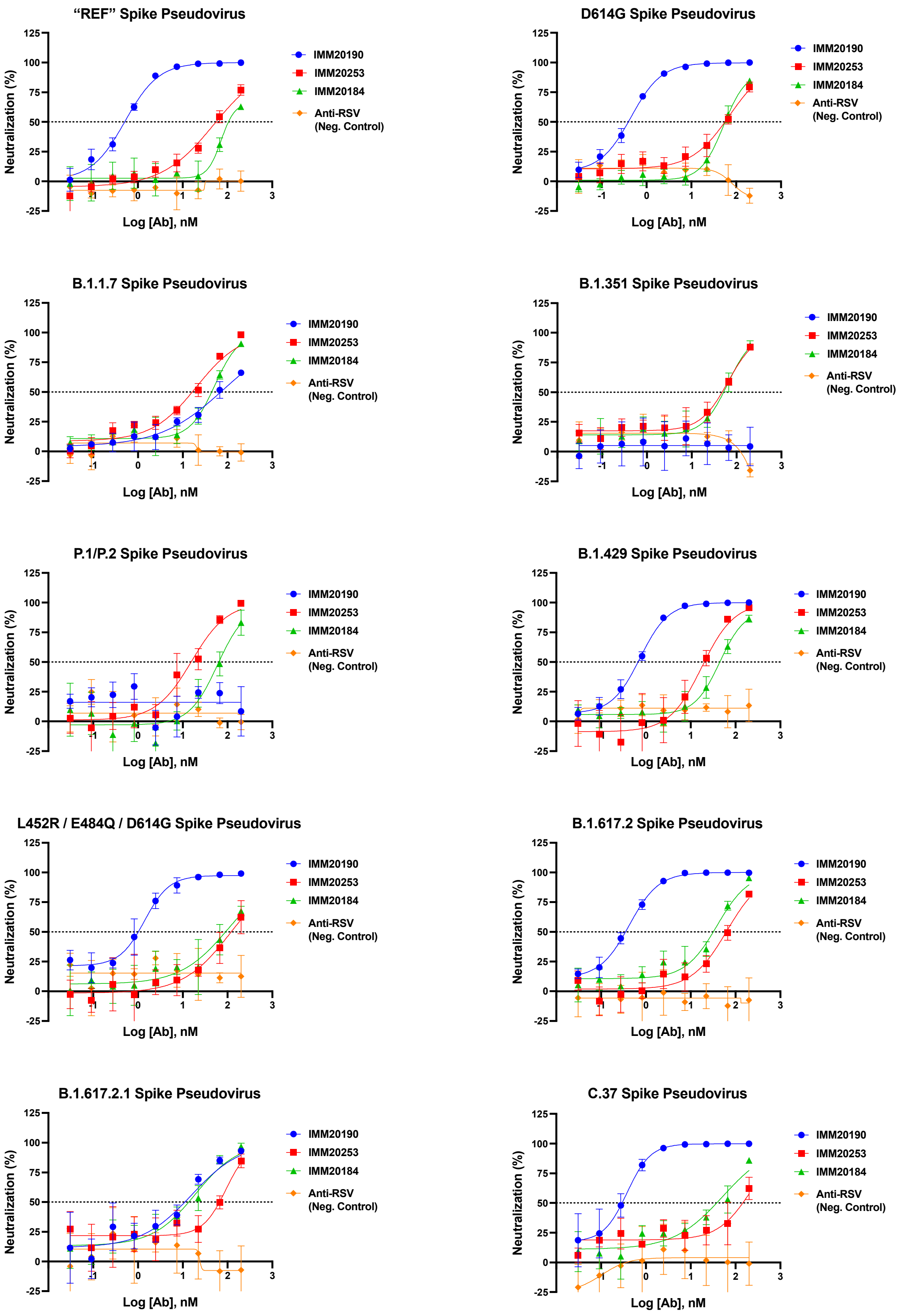
"REF" Spike Pseudovirus
D614G Spike Pseudovirus
B.1.1.7 Spike Pseudovirus
B.1.351 Spike Pseudovirus
P.1/P.2 Spike Pseudovirus
B.1.429 Spike Pseudovirus
L452R / E484Q / D614G Spike Pseudovirus
B.1.617.2 Spike Pseudovirus
B.1.617.2.1 Spike Pseudovirus
C.37 Spike Pseudovirus
Neutralization (%)
Log [Ab], nM
IMM20190
IMM20253
IMM20184
Anti-RSV
(Neg. Control)

B

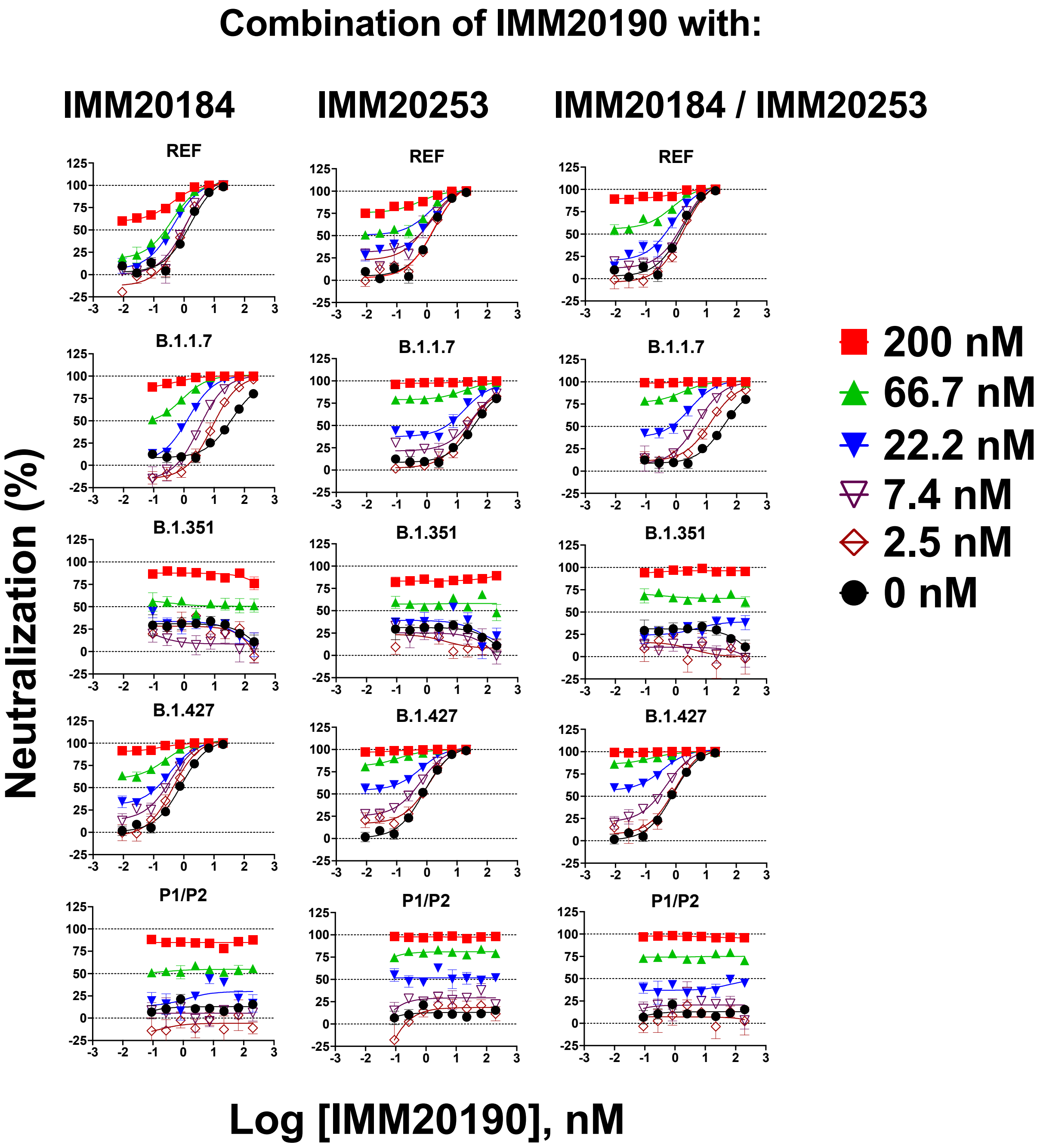
Combination of IMM20190 with:
IMM20184
IMM20253
IMM20184 / IMM20253
REF
B.1.1.7
B.1.351
B.1.427
P1/P2
Neutralization (%)
Log [IMM20190], nM
200 nM
66.7 nM
22.2 nM
7.4 nM
2.5 nM
0 nM

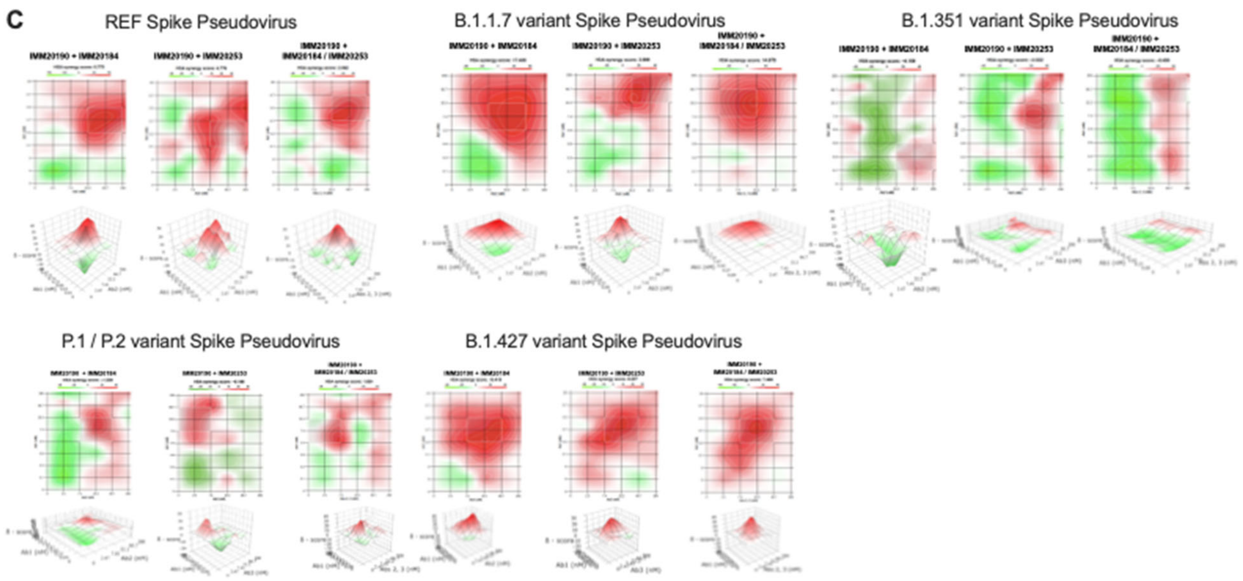

D

Variant	IMM20190 + IMM20184	IMM20190 + IMM20253	IMM20190 + IMM20184 / IMM20253	IMM20184 + IMM20253
Spike REF	5.8	4.8	2.1	
UK (B.1.1.7)	17.4	3.9	15.0	
SA (B.1.351)	- 4.1	- 3.0	- 8.4	2.53
Brazilian (P.1/P.2)	-1.23	-0.19	1.82	-3.35
California (B.1.429)	10.4	6.2	7.5	

Supplementary Figure 3. Three selected antibodies have a synergistic neutralizing effect. (A) Neutralization properties of standalone IMM20190, IMM20184 and IMM20253 antibodies against 10 different Spike variant pseudoviruses. (B) REF, B.1.1.7 (alpha), B.1.351 (beta), P1 (gamma), and B.1.427 (epsilon) pseudovirus variant neutralization by IMM20190 combination with either IMM20184, IMM20253 or both. (C) The Highest Single Agent (HSA) scores for 2-Ab and 3-Ab combinations. IMM20190 was mixed with IMM20184 and IMM20253 at 1:0.5:0.5 ratio. (D) HSA scores for two and three antibody cocktail.

Focus Reduction Neutralization Assay (FRNT)

A

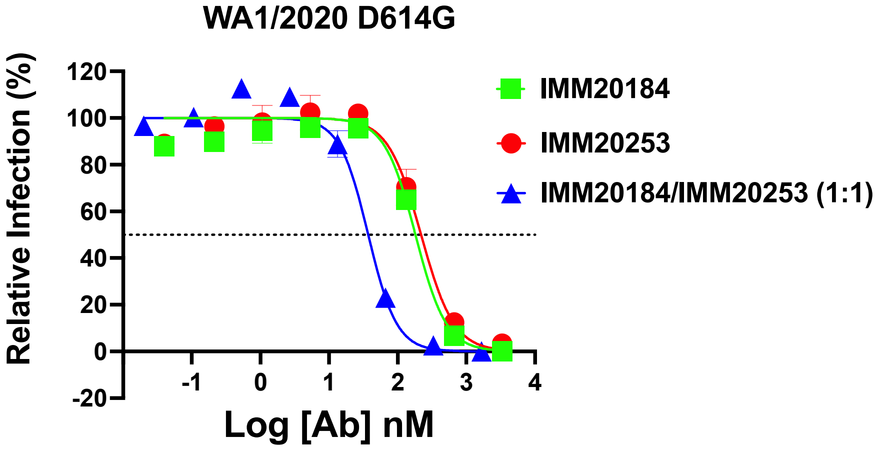

B

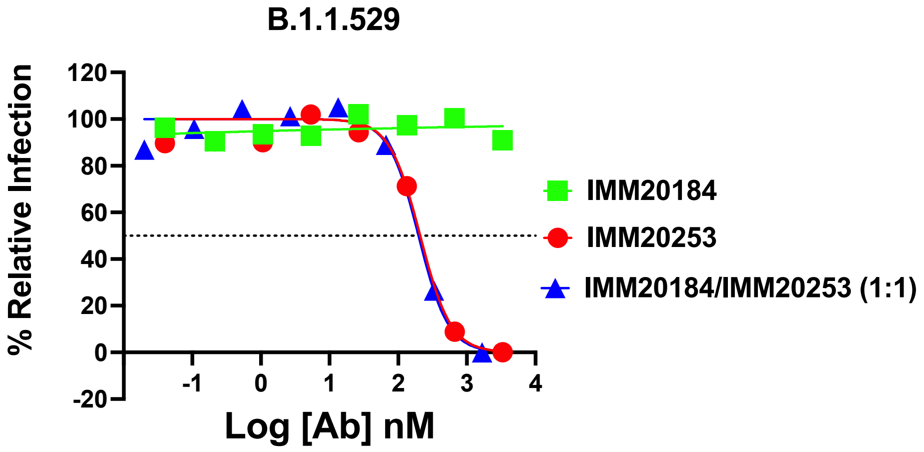

C

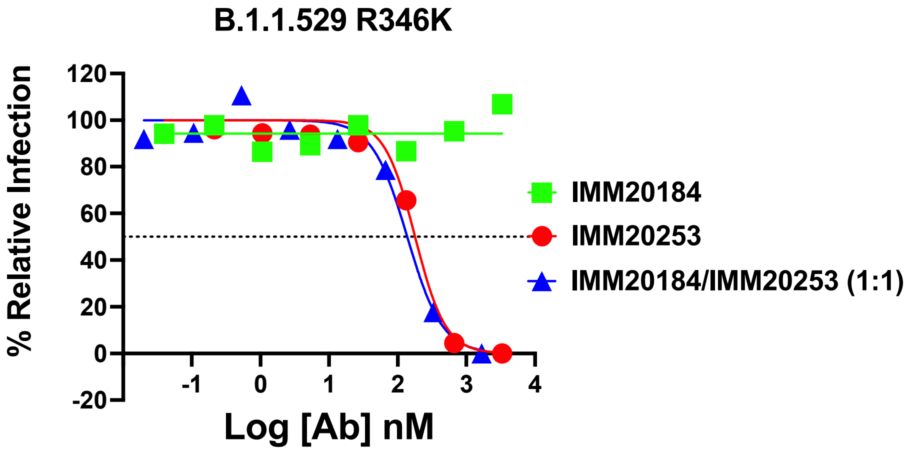

112

Supplementary Figure 4. Focus reduction neutralization assay (FRNT) of SARS-CoV-2 variants in the presence of IMM20184, IMM20253 and IMM20253/184 combination. (A) Relative infection of WA1/2020 D614G, (B) Omicron (BA.1) and (C) Omicron BA.1.1 virus variants in the presence of IMM20184, IMM20253 and IMM20184/253 antibodies**.** Data are representative of three independent experiments performed in duplicate.

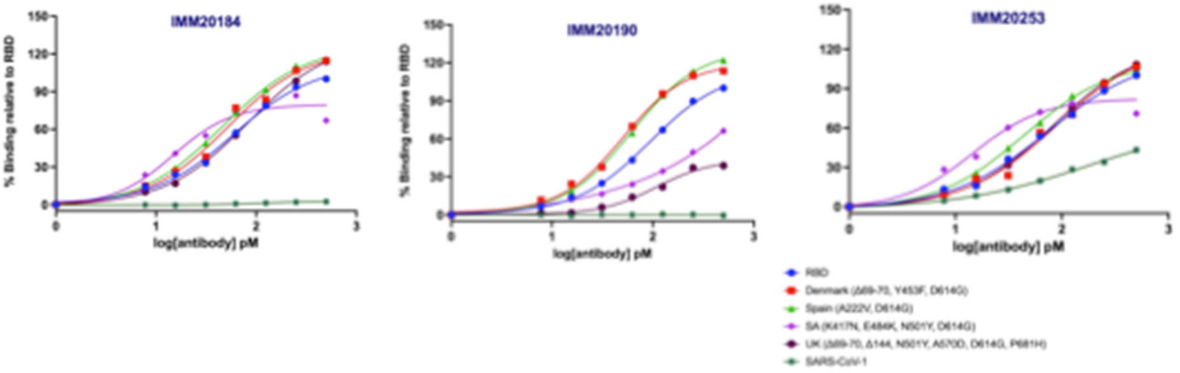

Supplementary Figure 5. Binding of IMM antibodies to soluble RBD proteins from SARS-CoV-1 and SARS-CoV-2 variants in a steady-state hTRF assay.
